# Cytotoxic Effects of Medicinal Plant Extracts on HeLa Cells via Redox Imbalance, L‐Arginine Metabolic Reprogramming, and Glycolytic Suppression

**DOI:** 10.1002/cbf.70307

**Published:** 2026-09-11

**Authors:** Mery Kocharyan, Gohar Sevoyan, Hasmik Karapetyan, Anzhela Sargsyan, Mikayel Ginovyan, Nikolay Avtandilyan, Hayarpi Javrushyan

**Affiliations:** ^1^ Research Institute of Biology Yerevan State University, Alex Manoogian Yerevan Armenia; ^2^ Laboratory of Immunology and Tissue Engineering of L.A. Orbeli Institute of Physiology of NAS RA Yerevan Armenia

**Keywords:** cytotoxicity, glycolysis, HeLa cells, l‐arginine metabolism, medicinal plant extracts, oxidative stress

## Abstract

Cancer cells undergo metabolic reprogramming to sustain proliferation, resist apoptosis, and adapt to oxidative stress. In the present study, we comparatively evaluated the anticancer effects of four medicinal plant extracts (*Inula helenium, Hypericum alpestre, Rumex obtusifolius*, and *Alchemilla smirnovii*) on HeLa cervical cancer cells, with particular emphasis on oxidative stress, l‐arginine metabolism, and cellular energy pathways. Cell viability was assessed using the MTT (3‐(4,5‐dimethylthiazol‐2‐yl)‐2,5‐diphenyltetrazolium bromide) assay, while biochemical colorimetric methods were used to determine nitric oxide production, arginase and nitric oxide synthase activities, malondialdehyde levels, superoxide dismutase and catalase activities, proline content, and key glycolytic metabolites. Nuclear morphology changes consistent with apoptotic features were examined by Hoechst 33258 staining. All tested extracts exhibited cytotoxic effects in HeLa cells in a time‐ and concentration‐dependent manner, which were further enhanced when combined with the metabolic inhibitors l‐NAME (Nω‐nitro‐l‐arginine methyl ester) or nor‐NOHA (Nω‐hydroxy‐nor‐l‐arginine). *Inula helenium* showed the strongest potentiation in combination treatments. Several extracts significantly decreased arginase activity while increasing nitric oxide synthase activity and nitrite levels, indicating modulation of l‐arginine metabolism. Treatments also increased malondialdehyde levels and stimulated antioxidant enzyme activities, consistent with redox imbalance and oxidative stress–mediated cytotoxicity. In addition, *Inula helenium* and *Alchemilla smirnovii* reduced intracellular glucose, pyruvate, and lactate concentrations, suggesting partial modulation of glycolytic metabolism. Fluorescence microscopy revealed chromatin condensation, nuclear fragmentation, and apoptotic body formation, particularly after treatment with *Hypericum alpestre* and *Rumex obtusifolius*. These findings demonstrate that selected medicinal plant extracts exert cytotoxic effects by simultaneously modulating oxidative stress, amino acid metabolism, and energy pathways in cervical cancer cells. The investigated plants may represent promising sources of bioactive compounds for the development of complementary metabolic anticancer strategies.

## Significance

This study highlights the cytotoxic activity of medicinal plant extracts by linking their phytochemical composition to functional effects in cancer cells. The results demonstrate that these extracts disrupt redox balance, alter l‐arginine metabolism, and suppress glycolytic activity, leading to reduced viability and induction of nuclear alterations consistent with apoptotic morphology in HeLa cells. By integrating biochemical assays with cellular responses, this work provides mechanistic insight into how plant‐derived compounds modulate key metabolic vulnerabilities of cancer cells. These findings support the potential of medicinal plants as a source of bioactive molecules for developing complementary anticancer strategies targeting metabolic reprogramming.

**Abbreviations**
5‐FU: 5‐fluorouracil
ARG: arginase
AS: *Alchemilla smirnovii* Juz
CAT: catalase
cGMP: cyclic guanosine monophosphate
CO_2_
: carbon dioxide
DNPH: 2,4‐dinitrophenylhydrazine
DW: dry weight
ECM: extracellular matrix
HA: *Hypericum alpestre*
IH: *Inula helenium*
IL‐2: interleukin‐2
l‐NAME: Nω‐nitro‐l‐arginine methyl ester
MDA: malondialdehyde
MTT: 3‐(4,5‐dimethylthiazol‐2‐yl)‐2,5‐diphenyltetrazolium bromide
NO: nitric oxide
nor‐NOHA: Nω‐hydroxy‐nor‐l‐arginine
NOS: nitric oxide synthase
PBS: phosphate‐buffered saline
RNS: reactive nitrogen species
RO: *Rumex obtusifolius*
ROS: reactive oxygen species
SD: standard deviation
SEM: standard error of the mean
sGC: soluble guanylate cyclase
SOD: superoxide dismutase
TBARS: thiobarbituric acid reactive substances
TCA: trichloroacetic acid

## 1 Introduction

Cancer remains a leading cause of morbidity and mortality worldwide, prompting intense research into the metabolic and molecular mechanisms that drive tumorigenesis and metastasis. Tumor cells differ fundamentally from normal cells in their energy requirements, biosynthetic demands, and ability to survive under stress, a distinction arising from genetic mutations, immune evasion, and the development of tolerance within the tumor microenvironment [1]. Metabolic reprogramming is therefore a hallmark of cancer, enabling malignant cells to meet their energy and nutrient needs while adapting to challenging microenvironmental conditions. Among the various pathways implicated, alterations in amino acid metabolism, particularly l‐arginine metabolism, have emerged as critical determinants of tumor progression and immune modulation [2]. Cervical cancer progression is strongly associated with oxidative stress imbalance, dysregulation of apoptotic pathways, and epigenetic alterations, including chromatin remodeling. Reactive oxygen species (ROS) can act as double‐edged mediators, promoting either tumor progression or apoptosis, depending on the cellular context and concentration.

l‐arginine is a multifunctional amino acid and a substrate for several key enzymes, including nitric oxide synthase (NOS) and arginase (ARG), which exist in multiple isoforms [1]. Arginine metabolism contributes to essential cancer processes: while NOS‐mediated conversion produces nitric oxide (NO), influencing signaling cascades that regulate tumor invasion and angiogenesis, arginase activity drives polyamine synthesis, promoting cellular proliferation and tumor growth [1]. This interplay is further modulated by cytokines such as IL‐2, which can upregulate arginine‐metabolizing enzymes and affect T cell function, highlighting the intimate link between cancer metabolism and immune regulation. Dysregulation of arginine metabolism can result in arginine depletion in the tumor microenvironment, impairing T cell proliferation and cytokine production, and ultimately facilitating immune evasion [3]. Targeted inhibition of these enzymes using agents such as l‐NAME (a NOS antagonist) and nor‐NOHA (an arginase inhibitor) has shown promise in limiting tumor growth and modulating immune responses [4, 5, 6]. Beyond amino acid metabolism, cancer cells exhibit global metabolic rewiring, exemplified by the Warburg effect, in which tumors preferentially metabolize glucose through aerobic glycolysis even in the presence of oxygen [7]. Although less efficient than oxidative phosphorylation at producing ATP, this pathway provides a rapid energy supply and generates biosynthetic precursors needed for proliferation [8]. Increased lactate production resulting from aerobic glycolysis not only supports tumor growth but also promotes angiogenesis and activates proinflammatory pathways, such as IL‐17 signaling, contributing to the establishment of a pro‐tumor microenvironment [9, 10].

Another characteristic feature of cancer is redox imbalance. Tumor cells generate persistently elevated levels of ROS, including superoxide anion (O_**2**_^**•−**^), hydroxyl radical (OH•), and hydrogen peroxide (H_2_O_2_) [11]. While moderate ROS levels function as signaling molecules regulating proliferation, differentiation, and immune responses, excessive ROS can induce oxidative damage, lipid peroxidation, and apoptosis [12]. The delicate balance between ROS production and antioxidant defenses is therefore critical for tumor survival and presents a potential therapeutic target. Plant‐derived compounds have been shown to modulate both ROS generation and antioxidant pathways, suggesting their potential as multi‐targeted anticancer agents [13].

The selected medicinal plants were chosen based on their documented traditional use and reported antioxidant, anti‐inflammatory, and potential anticancer properties. However, their direct mechanistic effects on cervical cancer cells remain insufficiently characterized. Recent studies have highlighted the promise of phyto‐therapeutic substances—including herbs, plant extracts, and natural compounds—as complementary or adjunctive cancer therapies. These agents can induce cytotoxicity in tumor cells, modulate cell metabolism, enhance immune signaling, and influence energy and redox pathways, offering a multi‐targeted approach to tumor control [14]. Despite these advances, understanding the precise mechanisms by which these compounds exert their effects, particularly in the context of combination treatments with enzyme inhibitors such as l‐NAME and nor‐NOHA, remains limited [6, 15].

Phytochemical profiling studies have demonstrated that medicinal plants are rich sources of biologically active compounds such as flavonoids, phenolic acids, terpenoids, and polyphenols, many of which exhibit pronounced antioxidant, anti‐inflammatory, and anticancer properties [6, 16, 17, 18]. Several of these compounds have been shown to interfere with key metabolic pathways involved in tumor progression, including glycolysis, amino acid metabolism, and redox regulation. Recent metabolomic and phytochemical analyzes using high‐performance liquid chromatography coupled with tandem mass spectrometry (HPLC–MS/MS) have provided detailed insights into the chemical composition of these plants and their potential bioactive constituents. Previous investigations of the selected plant species (*Hypericum alpestre*, *Rumex obtusifolius*, *Inula helenium*, and *Alchemilla smirnovii*) revealed the presence of phenolic compounds and other secondary metabolites with potential anticancer activity. However, despite increasing evidence of their pharmacological properties, the precise mechanisms by which these extracts influence cancer cell metabolism, oxidative stress balance, and arginine‐dependent signaling pathways remain insufficiently characterized [6, 15, 19, 20, 21, 22].

In this study, we investigated cytotoxic effects of a few herbal plants, including *Inula helenium, Hypericum alpestre*, *Rumex obtusifolius*, and *Alchemilla smirnovii* Juz—on HeLa cells, both individually and in combination with inhibitors of l‐arginine metabolism. By examining cytotoxic effects, changes in oxidative stress markers, and impacts on arginine‐related enzymatic pathways, we aimed to elucidate the mechanistic basis for their potential anticancer activity. Our findings provide further insight into the potential role of natural compounds in modulating cancer metabolism, oxidative balance, and immune responses, and support further investigation of plant‐derived compounds in combination with conventional chemotherapeutic agents.

## 2 Materials and Methods

### 2.1 Chemicals and Reagents

All chemicals and reagents were purchased from Sigma‐Aldrich GmbH (Taufkirchen, Germany).

### 2.2 Plant Material: Collection, Identification, and Extraction

The plants *Hypericum alpestre* (HA; aerial parts), *Inula helenium* (IH; flowers and leaves), *Rumex obtusifolius* (RO; seeds), and *Alchemilla smirnovii* Juz. (AS; aerial parts) were harvested from the Tavush region of Armenia at altitudes of 1450–2400 m above sea level. Aerial parts were collected during the flowering period (June–July), while seeds were collected after maturation (August). Plant identification was carried out at the Department of Botany and Mycology, Yerevan State University (Armenia) by Dr. Narine Zakaryan. Voucher specimens were deposited in the Herbarium of Yerevan State University, where voucher numbers were assigned [19, 21, 22]. Crude plant extracts (50 mg DW/mL) were prepared by maceration using 96% ethanol at a solvent‐to‐sample ratio of 10:1 (vol/wt). The percentage yield of the extracts had been determined previously. For cell‐based assays, the crude extracts were diluted to final concentrations of 0.125, 0.25, and 0.5 mg DW/mL in culture medium. The phytochemical composition of all the investigated plant extracts has been previously characterized using high‐performance liquid chromatography coupled with tandem mass spectrometry (HPLC–MS/MS), which identified multiple phenolic compounds and secondary metabolites potentially responsible for their biological activity. Detailed phytochemical profiles are available in our previous publications [15, 19, 20, 22].

### 2.3 LC‐Q‐Orbitrap‐HRMS in Plant Extracts

The studied plant materials included AS (voucher No. ERCB13638), IH (ERCB13919), RO (ERCB13208), and HA (ERCB13206). Voucher specimens were deposited in the Herbarium of Yerevan State University. The phytochemical composition of the extracts was characterized using ultra‐high‐performance liquid chromatography coupled with high‐resolution mass spectrometry (UHPLC–HRMS). Qualitative analysis was performed on a Dionex UltiMate 3000 UHPLC system coupled to a Q Exactive Focus quadrupole‐Orbitrap mass spectrometer equipped with a heated electrospray ionization (HESI‐II) source, operating in negative ion mode. Semi‐quantitative analysis of major phenolic constituents was performed using LC–PDA with external calibration, and results were expressed either as absolute concentrations or as equivalents of structurally related standards. Detailed chromatographic conditions, compound identification data, and quantitative profiles are provided in Supporting Information Files S1 and S2.

### 2.4 Cell Culture

The human cervical cancer cell line HeLa (Homo sapiens, female, cervix adenocarcinoma; ATCC CCL‐2) was used in this study. Cells were obtained from the Institute of Physiology (Yerevan, Armenia), which originally sourced the cell line from the American Type Culture Collection (ATCC, Manassas, VA, USA). Cells were cultured in Dulbecco's Modified Eagle Medium supplemented with 10% fetal bovine serum and 1% penicillin–streptomycin under standard conditions (37°C, 5% CO_2_) (Biosan S‐Bt Smart Biotherm, Latvia) [20]. The identity of the cell line was verified by the original supplier (ATCC), and cells were handled according to standard cell culture practices. The cell line has not been reported as misidentified or contaminated in our laboratory. Cultured cells were regularly examined for the presence of mycoplasma contamination using the Universal Mycoplasma Detection Kit (ATCC, Manassas, VA, USA), and all experiments were performed using mycoplasma‐free cells.

### 2.5 MTT Cytotoxicity Assay

Cell viability was evaluated using the MTT assay to assess growth inhibition in HeLa cells exposed for 4, 24, or 72 h to different concentrations (0.5, 0.25, and 0.125 mg DW/mL) of HA, IH, and AS extracts, as well as the inhibitors l‐NAME (14 µM), nor‐NOHA (10 µM), and 5‐fluorouracil (5‐FU; 40 µM) [19]. Each treatment was performed in three technical replicates, and three independent biological experiments were conducted. Cytotoxicity was expressed as the percentage of growth inhibition relative to control cells treated with the appropriate volume of solvent only (1% ethanol in the final mixture), whose growth was considered 100%.

### 2.6 Treatment of Cells for Biochemical Analyzes

HeLa cells were seeded in 24‐well plates (5 × 10^4^ cells per well) and incubated for 24 h. The culture medium (450 µL) was then refreshed, and cells were treated with 50 µL of control or test compounds to achieve the following final concentrations: PBS, 1% ethanol (control), 5‐FU (40 µM), l‐NAME (14 µM), nor‐NOHA (10 µM), AS (0.125 and 0.25 mg DW/mL), RO (0.125 and 0.25 mg DW/mL), HA (0.125 and 0.25 mg DW/mL), and IH (0.125 and 0.25 mg DW/mL) [23]. After 24 h incubation, the culture medium was discarded, and cells were collected by trypsinization, neutralization, and centrifugation. Cell pellets were lysed on ice using lysis buffer and further incubated for 10 min. After centrifugation at 13,000 *g* for 10 min at 4°C, the supernatants were collected. Nitrite anions, MDA, arginase, NOS, SOD, catalase, glucose, pyruvate, lactate, and proline were quantified as described below. The selected extract concentrations were based on preliminary dose–response experiments and corresponded to sub‐cytotoxic or moderately cytotoxic concentrations suitable for investigating metabolic and biochemical alterations in cancer cells.

### 2.7 Determination of Nitric Oxide (NO) Levels

NO production was assessed by measuring nitrite anions using the Griess reaction. For each sample, 100 µL was mixed with 100 µL of Griess reagent. Supernatants were incubated with cadmium pellets at room temperature for 12 h to convert nitrate to nitrite. Absorbance was measured at 550 nm, and NO concentration was calculated using a sodium nitrite standard curve [19].

### 2.8 Determination of Malondialdehyde (MDA)

MDA levels were determined using the thiobarbituric acid‐reactive substances (TBARS) assay according to the Ohkawa method. Absorbance was measured spectrophotometrically, and MDA concentration was calculated based on a standard curve [20].

### 2.9 Determination of Superoxide Dismutase (SOD) Activity

SOD activity was determined by measuring the inhibition of adrenaline auto‐oxidation. One unit of SOD activity was defined as the amount of enzyme required to inhibit the reaction rate by 50% and expressed as conventional units [6].

### 2.10 Determination of Catalase Activity

Catalase activity was measured by monitoring the decrease in hydrogen peroxide (H_2_O_2_) concentration at 374 nm. The reaction was stopped with 4% ammonium molybdate, forming a stable yellow complex. Catalase activity was expressed as µmol of H_2_O_2_ decomposed per minute per milliliter of cell lysate [6].

### 2.11 Determination of Proline Content

Proline levels were determined in cell lysates incubated with ornithine, α‐ketoglutarate, and pyridoxal‐5‐phosphate at 37°C for 1 h. The reaction was stopped with trichloroacetic acid (TCA), centrifuged, and the proline concentration was determined colorimetrically. Results were expressed as µmol proline/g protein [24].

### 2.12 Determination of Glucose Concentration

Glucose concentration was measured using an enzymatic glucose oxidase–peroxidase colorimetric method. Absorbance was recorded at 505 nm, and glucose levels were calculated from a standard curve prepared with d‐glucose [25].

### 2.13 Determination of Pyruvate Concentration

Pyruvate concentration was determined using a colorimetric assay based on its reaction with 2,4‐dinitrophenylhydrazine (DNPH), forming a hydrazone complex measured spectrophotometrically at 520 nm. Pyruvate concentration was calculated using a sodium pyruvate standard curve [26].

### 2.14 Determination of Lactate Concentration

Lactate levels were determined using an enzymatic lactate oxidase‐based colorimetric assay, in which hydrogen peroxide generated during lactate oxidation reacts with a chromogenic substrate. Absorbance was measured at 540 nm, and lactate concentration was calculated using a lactate standard curve [27].

### 2.15 Determination of Arginase Activity

Arginase activity in cell lysates was measured using the modified diacetyl monoxime colorimetric method and expressed as enzyme activity per milligram of protein [19].

### 2.16 Determination of Nitric Oxide Synthase (NOS) Activity

NOS activity was assessed by measuring the conversion of l‐arginine to l‐citrulline. Total, Ca^2+^‐dependent, and Ca^2+^‐independent NOS activities were determined as described previously. Results were normalized to protein content [19].

### 2.17 Analysis of Nuclear Morphology Changes by Hoechst 33258 Staining

Nuclear morphological changes were assessed by Hoechst 33258 staining as previously described [20], where this method yielded results comparable to those obtained by ELISA and Western blot analyzes of caspase‐3 activity for apoptosis evaluation. HeLa cells (2 × 10^5^ cells/mL) were treated for 24 h with vehicle, 5‐FU (40 μM), RO (0.25 mg/mL), or HA (0.25 mg/mL). After treatment, the cells were washed with PBS and fixed with 4% paraformaldehyde in PBS for 10 min. The cells were then washed twice with PBS for 5 min and stained with Hoechst 33258 reagent (10 μg/mL) for 10 min at room temperature in the dark. After staining, the cells were washed with PBS and examined under a fluorescence microscope (Zeiss, Germany) at ×250 magnification. Fluorescence images were acquired using a DAPI filter set with excitation at 350‐365 nm and emission at 450–470 nm. Cells exhibiting hyperfluorescent nuclei or condensed chromatin, nuclear fragmentation, and/or apoptotic body‐like structures were classified as showing apoptosis‐like nuclear morphology. All treatment variants were examined in duplicate. For each treatment variant, 500 cells were scored, and the percentage of cells with apoptosis‐like nuclear morphology was calculated as follows: % of cells with apoptosis‐like nuclear morphology = (number of cells with apoptosis‐like nuclear morphology/500 cells) × 100.

### 2.18 Statistical Analysis

Results are presented as mean ± standard deviation (SD). Statistical analyzes were performed using GraphPad Prism 8 (San Diego, CA, USA). Differences were considered statistically significant at *p* < 0.05.

## 3 Results

### 3.1 Effects of Treatments on HeLa Cell Viability

Comprehensive phytochemical profiling using UHPLC–HRMS revealed a complex composition of phenolic compounds and secondary metabolites across all studied plant extracts. High‐resolution mass spectrometry enabled the annotation of major constituents based on accurate mass and diagnostic MS/MS fragmentation patterns, confirming the presence of flavonoids, phenolic acids, and tannin‐related compounds. Semi‐quantitative analysis further demonstrated notable differences in the content of key metabolites among the investigated species, reflecting distinct chemical profiles. The full list of identified compounds, along with their retention times, mass spectral data, and quantitative values, is presented in Supporting Information Files S1 and S2. These findings highlight the chemical diversity of the extracts and provide a basis for the differential biological activity observed in subsequent cellular assays.

**Figure 1:**
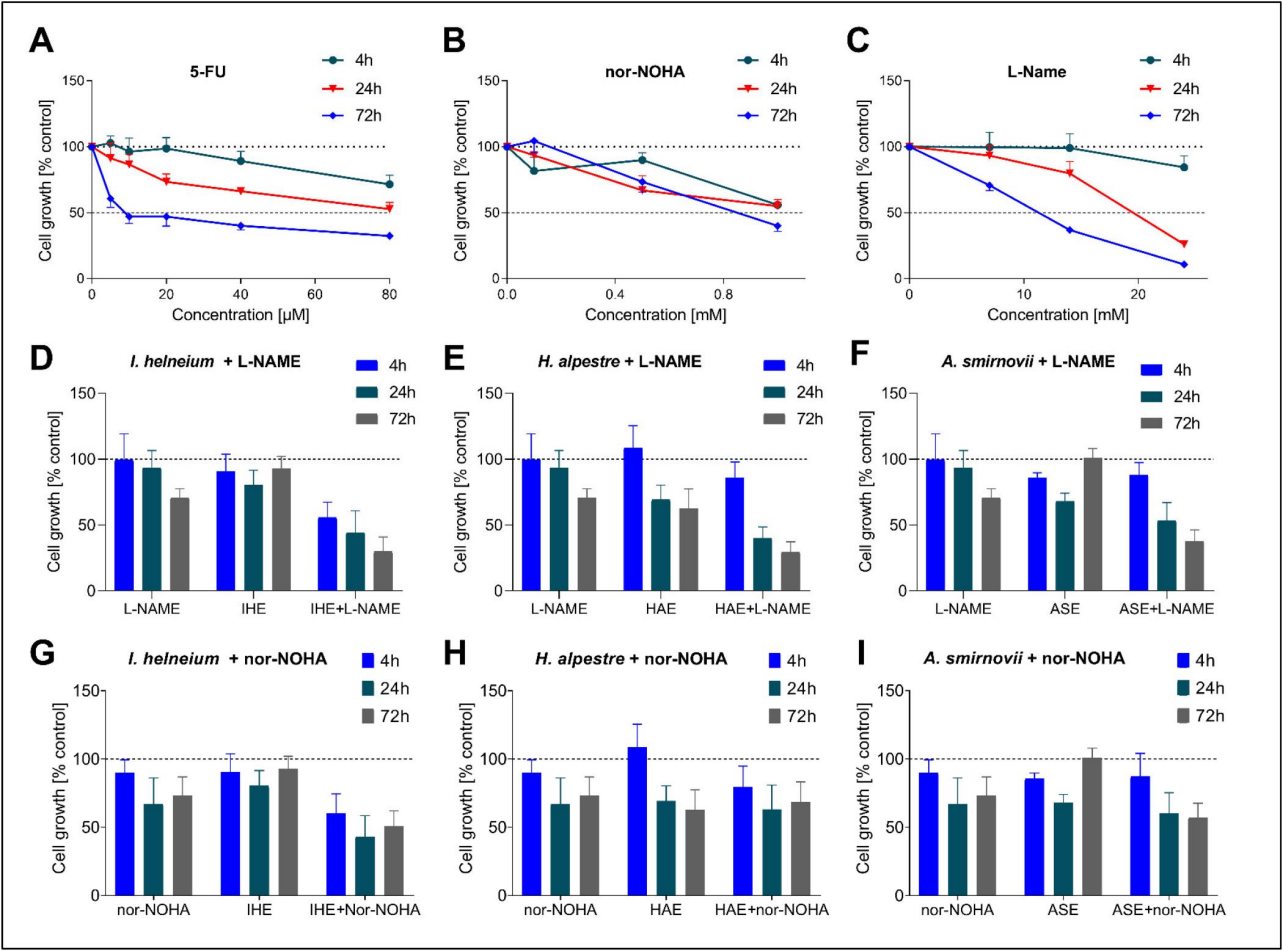
Cytotoxic effects of chemotherapeutic agents (A, B, and C) and combined treatment with plant extracts and chemotherapeutic agents (D–I) on HeLa cells. Cells were treated with 5‐fluorouracil (5‐FU, 0–80 µM) (A), nor‐NOHA (0–1 mM) (B), or l‐NAME (0–24 mM) (C) for 4, 24, and 72 h. Cells were treated with *Inula helenium* (IH), *Hypericum alpestre* (HA), or *Alchemilla smirnovii* (AS) extracts (0.25 mg DW/mL) in combination with nor‐NOHA (0.5 mM) (G–I) or l‐NAME (14 mM) (D–F) for 4, 24, and 72 h. Cell viability was assessed using the MTT assay and expressed as a percentage of control cells. Data represent mean ± SD (*n* = 3). Differences were considered statistically significant at *p* < 0.05.

The cytotoxic effects of synthetic compounds and plant extracts on HeLa cells were assessed using the MTT assay after 4, 24, and 72 h of exposure. Treatment with the chemotherapeutic agent 5‐fluorouracil (5‐FU, 0–80 µM) resulted in a time‐dependent decrease in cell viability, with a pronounced cytotoxic effect observed at 72 h compared with earlier time points. Nor‐NOHA (0–1 mM), an arginase inhibitor, showed moderate cytotoxicity, which became statistically significant mainly at 72 h. In contrast, l‐NAME (0–24 mM), a nitric oxide synthase inhibitor, induced significant cytotoxic effects already at 24 h, which were further enhanced at 72 h (Figure 1). Notably, compared with 5‐FU, l‐NAME exhibited an earlier onset of cytotoxicity and a more pronounced reduction in cell viability at 24 h, whereas 5‐FU showed significant effects predominantly at 72 h.

The combined effects of plant extracts with arginase and NOS inhibitors were further investigated to assess their potential to enhance cytotoxic responses in HeLa cells (Figure 1D–I). When applied individually at sub‐inhibitory concentrations, *Inula helenium* (IH), *Hypericum alpestre* (HA), and *Alchemilla smirnovii* Juz. (AS) extracts produced moderate reductions in cell viability at 24 and 72 h. However, combination treatment with nor‐NOHA or l‐NAME resulted in a markedly greater cytotoxic effect than either treatment alone. Notably, the combination of IH with nor‐NOHA induced the greatest reduction in cell viability, achieving approximately 60% growth inhibition after 24 h of treatment (Figure 1G). In comparison, combinations of HA or AS with nor‐NOHA resulted in growth inhibition of no more than 50% (Figure 1H,I). Treatment with l‐NAME, combined with all three plant extracts, led to a substantial increase in cytotoxicity, particularly at 24 and 72 h of exposure. At 24 h, IH combined with l‐NAME reduced cell viability by approximately 60% (Figure 1D), while HA + l‐NAME and AS + l‐NAME resulted in approximately 40% and 50% growth inhibition, respectively (Figure 1E,F). Treatment with HA alone did not induce significant cytotoxicity in HeLa cells (Figure 1E). On the contrary, a slight stimulation of cell proliferation was observed at 4 h of exposure; however, at later time points (24 and 72 h), cell viability declined gradually, although these changes did not reach statistical significance. A similar trend was observed for AS administered alone. This extract did not demonstrate a pronounced cytotoxic effect at either 4 or 24 h. In contrast, a modest increase in cell viability was observed after 72 h of treatment, suggesting a time‐dependent proliferative response rather than direct cytotoxicity. These observations suggest that the modulation of l‐arginine metabolism may sensitize cancer cells to the cytotoxic effects of plant‐derived bioactive compounds, highlighting the potential synergistic interaction between metabolic inhibitors and phytochemicals.

### 3.2 Modulation of l‐Arginine Metabolism by Chemotherapeutic Agents and Plant Extracts

To investigate the effects of plant extracts and chemotherapeutic agents on l‐arginine metabolism, key components of this pathway—arginase activity, nitric oxide synthase (NOS) activity, and nitrite ion (Nitric oxide) levels—were evaluated. In all experiments, plant extracts were applied at 0.25 mg DW/mL and 0.125 mg DW/mL, while 5‐FU was used at 40 μM. The selected plant concentrations were based on prior MTT assay results, and the inclusion of 5‐FU reflects its established role as a reference anticancer drug [19, 21].

**Figure 2:**
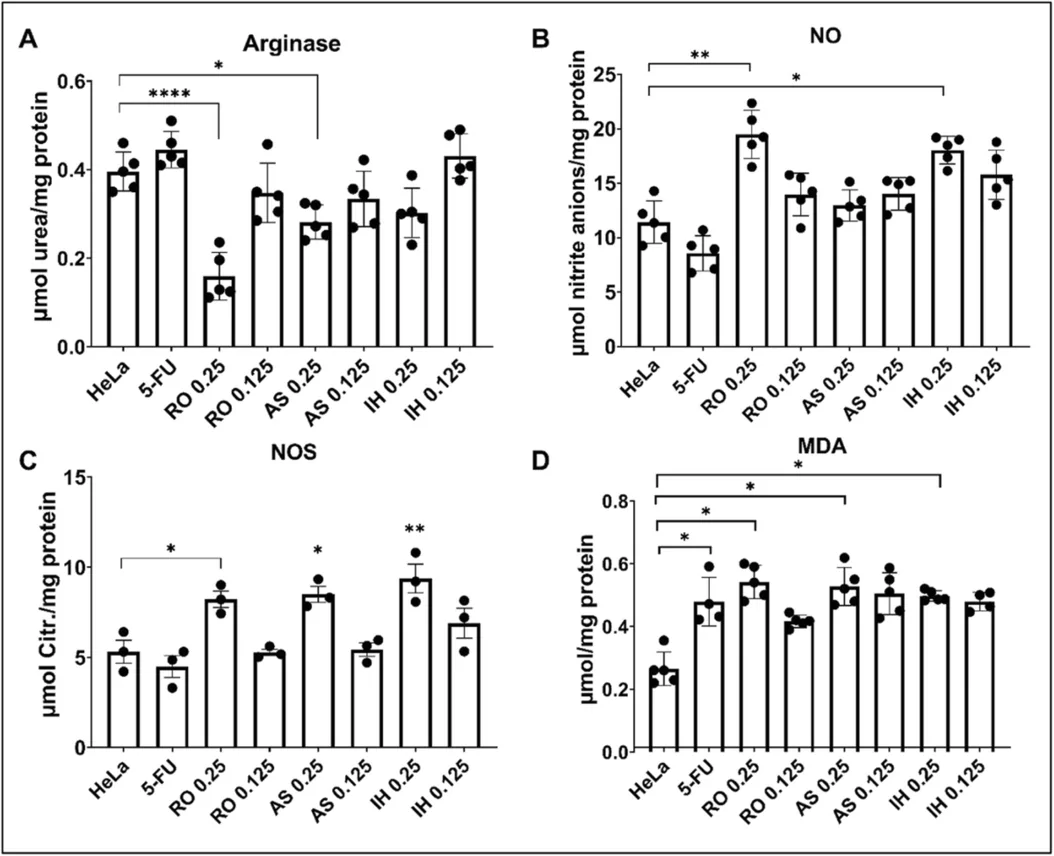
Effects of plant extracts and 5‐fluorouracil (5‐FU) on l‐arginine metabolism and oxidative stress markers in HeLa cells. (A) Changes in arginase activity (*n* = 5). (B) Quantitative changes in nitric oxide (NO), expressed as nitrite concentration (*n* = 5). (C) Changes in nitric oxide synthase (NOS) activity (*n* = 3). (D) Levels of malondialdehyde (MDA) (*n* = 5). Cells were treated with plant extracts and 5‐FU as indicated. Data are presented as mean ± SEM. Statistical significance was determined relative to the control group (*p < 0.05; **p < 0.01, ****p < 0.0001).

Assessment of arginase activity revealed that treatment with plant extracts influenced this enzyme in a concentration‐dependent manner. Specifically, compared with the control group, a significant reduction in arginase activity was observed at the higher concentration (0.25 mg DW/mL) of RO and AS. Under these conditions, arginase activity decreased by approximately 2.2‐fold (****p < 0.0001 for RO) and 25% (*p < 0.05 for AS), respectively (Figure 2A). In contrast, treatment with 5‐FU alone did not induce statistically significant changes in arginase activity; however, the data indicate a tendency toward increased arginase activity compared with the control.

NOS activity, evaluated as an indicator of nitric oxide (NO) synthesis, was significantly elevated following treatment with IH, RO, and AS at a concentration of 0.25 mg DW/mL. Under these conditions, NOS activity increased by approximately 1.7‐fold compared with the control group (*p < 0.05), suggesting enhanced NO‐generating capacity (Figure 2B). In contrast, neither 5‐FU nor lower concentrations of the mentioned plant extracts induced a significant elevation in NOS activity. Alterations in nitrite ion levels were notable: treatment with IH extract at both tested concentrations resulted in a pronounced increase (*p < 0.05 for IH 0.25 mg DW/ml). AS extract also elevated nitrite ion levels, although to a slightly lesser extent than Inula helenium, but still markedly higher than with 5‐FU treatment. RO extract (0.25 mg DW/ml) significantly increased the quantity of nitrite anions in the cell medium (Figure 2B).

### 3.3 Redox Imbalance Induced by Treatments in HeLa Cells

**Figure 3:**
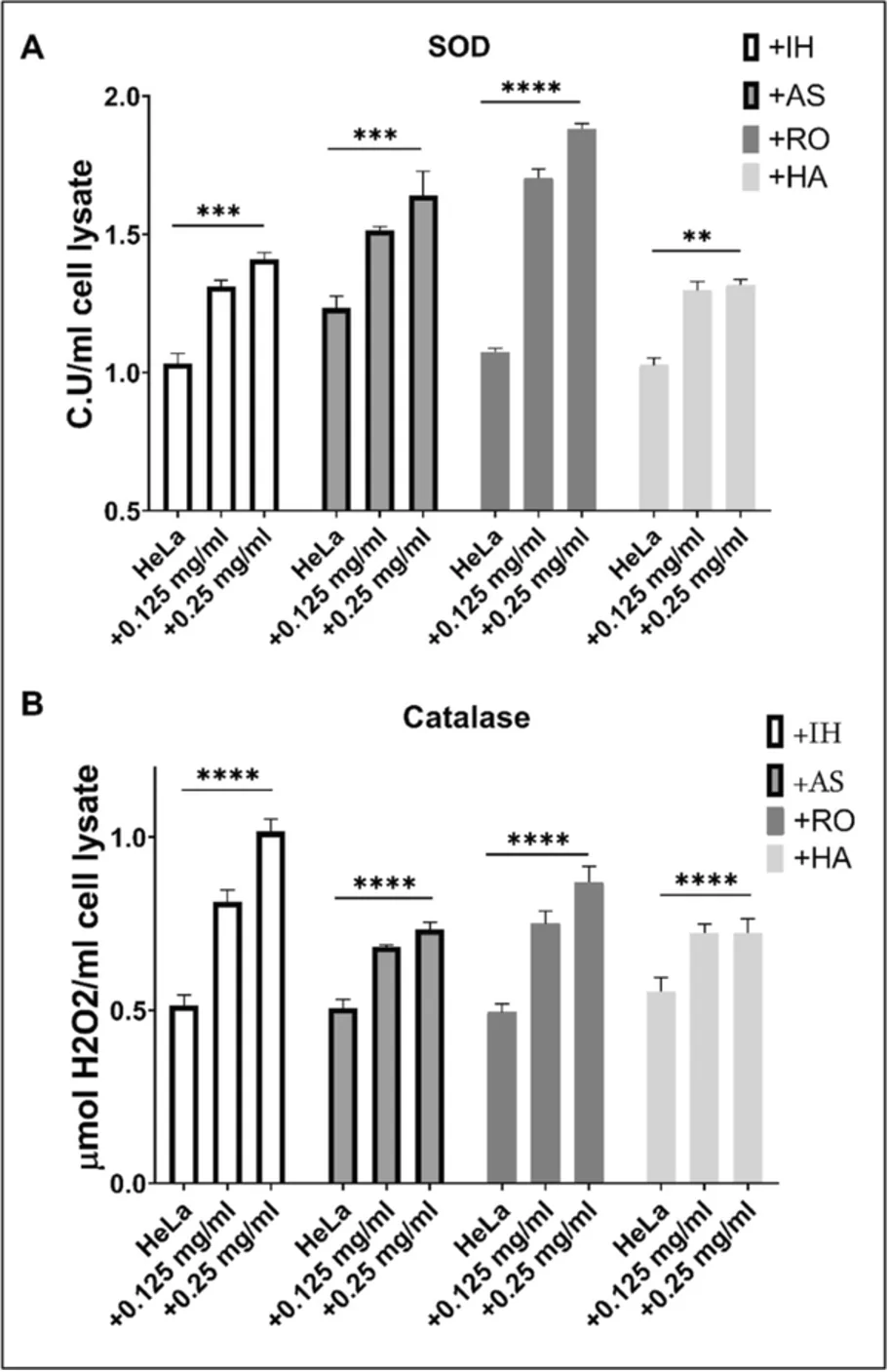
Effects of plant extracts on superoxide dismutase (SOD) (A) and catalase (CAT) (B) activity in HeLa cells. Cells were treated for 24 h with IH, AS, RO, and HA at concentrations of 0.25 and 0.125 mg DW/mL. Data are presented as mean ± SD (*n* = 3). Statistical significance was determined relative to the control group (****p < 0.0001; ***p < 0.001; **p < 0.01).

Since alterations in l‐arginine metabolism can influence cellular oxidative status, we next evaluated markers of redox balance in HeLa cells following treatment with selected plant extracts at the 24‐h time point. Specifically, lipid peroxidation and key antioxidant enzymes were assessed by measuring malondialdehyde (MDA) levels (Figure 2D), superoxide dismutase (SOD) activity (Figure 3A), and catalase activity (Figure 3B) to determine how these treatments affect cellular oxidative stress.

MDA is a widely used indicator of lipid peroxidation and oxidative stress. Quantitative analysis revealed that, compared with the control group, all tested plant extracts and 5‐FU increased MDA levels. In particular, treatment with 5‐FU resulted in an approximately twofold elevation in MDA content (*p* < 0.05). Plant extracts at a concentration of 0.25 mg DW/mL induced a more moderate but significant increase in MDA levels, ranging from approximately 1.3‐ to 1.5‐fold relative to the control group (*p* < 0.05) (Figure 2D). These changes in lipid peroxidation provided a rationale for further evaluation of enzymatic antioxidant responses.

The effects of plant extracts (IH, AS, RO, and HA) on intracellular antioxidant enzyme activity in HeLa cells are presented in Figure 3. Treatment with all tested extracts significantly increased SOD activity compared with untreated HeLa cells (Figure 3A). Both concentrations (0.125 and 0.25 mg/mL) enhanced enzyme activity, demonstrating a dose‐dependent trend. IH treatment significantly elevated SOD levels at both concentrations (***p < 0.001), with the higher dose showing a greater increase. A similar pattern was observed for AS (***p < 0.001), indicating strong stimulation of the antioxidant defense system. RO treatment produced the most pronounced increase in SOD activity (****p < 0.0001), particularly at 0.25 mg/mL. HA also significantly increased SOD activity (*p* < 0.01), although the effect was relatively modest. Overall, RO exhibited the strongest SOD‐inducing effect, followed by AS and IH, while HA showed a milder but still significant response. Catalase activity was also significantly increased in all treated groups compared to control cells (Figure 3B). Similar to SOD, CAT activity demonstrated a dose‐dependent elevation. IH significantly increased CAT levels (****p < 0.0001), with the higher concentration producing the greatest effect. AS treatment resulted in a marked elevation of CAT activity (****p < 0.0001).

RO treatment also significantly increased CAT levels (****p < 0.0001), confirming its strong capacity to modulate antioxidant activity. HA also significantly increased catalase activity (****p < 0.0001), although to a slightly lesser extent than RO. These findings indicate that all tested plant extracts significantly stimulate endogenous antioxidant defense mechanisms in HeLa cells by upregulating SOD and CAT activity. The effects were concentration‐dependent, suggesting that higher doses provide stronger antioxidant responses. Among the extracts, RO demonstrated the most potent antioxidant‐enhancing activity.

Such coordinated changes in lipid peroxidation and antioxidant enzyme activity indicate an altered redox state that may contribute to apoptosis‐associated cellular stress.

### 3.4 Changes in Proline Levels Associated With Altered Arginase Activity

**Figure 4:**
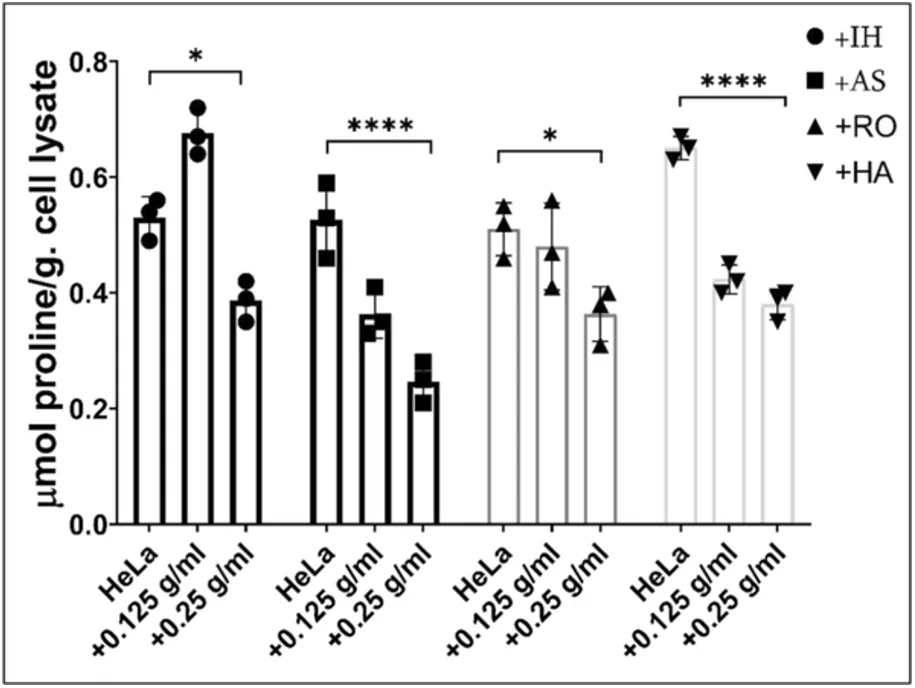
Effects of plant extracts on proline biosynthesis in HeLa cells. Cells were treated with IH, AS, RO, and HA at concentrations of 0.25 and 0.125 mg DW/mL. Proline biosynthesis was assessed at the 24‐h time point. Data are presented as mean ± SD (*n* = 3). Statistical significance was determined relative to the control group (****p < 0.0001; ***p < 0.001; **p < 0.01; *p < 0.05).

Amino acids, particularly proline, play a crucial role in cancer cell metabolism and growth. To assess the effect of selected plant extracts on proline metabolism, quantitative changes in proline levels were measured in HeLa cell lysates following treatment with plant extracts at concentrations of 0.125 and 0.25 mg DW/mL (Figure 4). Treatment with the tested extracts significantly altered intracellular proline levels compared with untreated control cells, with effects varying by extract and concentration. In the case of IH, proline levels showed a biphasic, concentration‐dependent pattern: the lower dose (0.125 mg/mL) tended to increase proline content relative to untreated cells, whereas the higher dose (0.25 mg/mL) reduced it below baseline (*p < 0.05; Figure 4). In contrast, AS treatment significantly decreased proline levels in a dose‐dependent manner (****p < 0.0001), with the most pronounced reduction observed at 0.25 mg/mL. Similarly, RO treatment caused a significant decrease in proline content (*p < 0.05), particularly at the higher concentration. HA treatment also significantly reduced intracellular proline levels (****p < 0.0001), with both concentrations showing lower levels than in untreated cells. Overall, while IH at the lower concentration slightly increased proline accumulation, most extracts—particularly AS and HA—significantly reduced intracellular proline levels in HeLa cells. The observed decrease in proline may reflect modulation of cellular redox balance and stress‐response pathways, suggesting that these extracts influence metabolic adaptation mechanisms in cancer cells.

### 3.5 Alterations in Cellular Energy Metabolism

**Figure 5:**
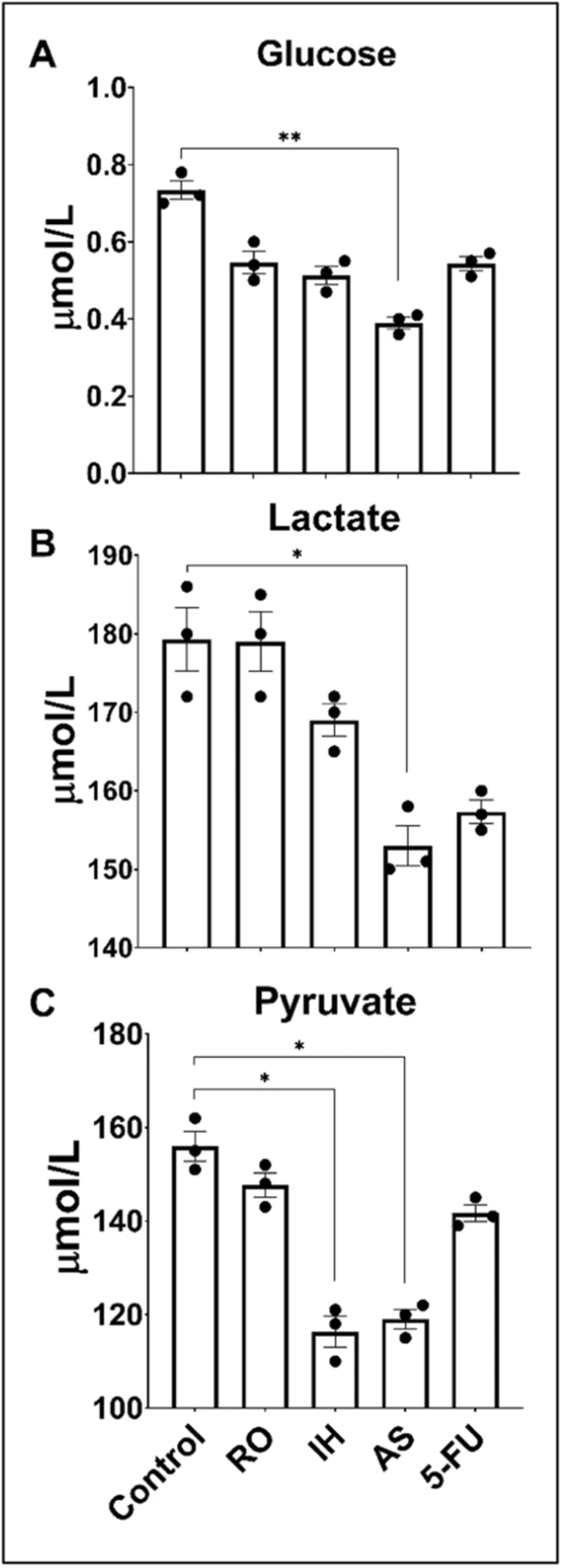
Effects of plant extracts and 5‐FU on key glycolytic intermediates in HeLa cells. Levels of glucose (A), lactate (B), and pyruvate (C) were measured after treatment with RO, IH, AS (0.25 mg DW/mL), and 5‐FU (40 µM). Data are presented as mean ± SD. Statistical significance was determined relative to the control group (**p < 0.01; *p < 0.05).

The observed alterations in glycolytic intermediates suggest that selected plant extracts modulate cellular energy metabolism in HeLa cells, whereas 5‐FU alone had minimal impact. HA did not show substantial glycolytic changes and was excluded from Figure 5. RO was retained in this analysis because it produced measurable changes in the evaluated glycolytic intermediates, although these effects were less pronounced than those observed with AS and IH.

Given that energy metabolism is closely connected to amino acid utilization and redox balance, these metabolic changes may contribute to the broader effects of the treatments on cell viability and proline biosynthesis observed in previous experiments. To evaluate the effects of plant extracts and 5‐FU on cellular energy metabolism, quantitative changes in key glycolytic intermediates—glucose, pyruvate, and lactate—were assessed in HeLa cells. Among the tested plants, AS and IH demonstrated the most pronounced effects on these metabolites.

Compared with the control group, glucose levels decreased by approximately 48% under AS treatment, whereas IH and other treatments produced a more moderate reduction of 28%–30% (***p < 0.001; Figure 5A). Lactate levels were also affected, with a decrease of approximately 16% under AS treatment and 7% under IH treatment (*p < 0.05; Figure 5B). Pyruvate content was reduced by 27% and 25% under AS and IH treatment, respectively, compared with control cells (*p < 0.05; Figure 5C). RO extract produced comparatively modest changes in glycolytic intermediates, with a moderate reduction in glucose levels (~25%) and a slight decrease in pyruvate content (~5%) relative to untreated cells, while lactate levels remained essentially unchanged. Unlike IH and AS, none of these RO‐induced alterations reached statistical significance under the tested conditions (Figure 5A–C). All experiments were conducted using a plant extract concentration of 0.25 mg/mL after 24 h of treatment. In contrast, treatment with 5‐FU alone did not produce significant changes in glucose, lactate, or pyruvate levels, indicating that, under these conditions, the chemotherapeutic drug had a limited effect on the energy metabolism of HeLa cells.

These findings suggest that certain plant extracts may interfere with the glycolytic phenotype typical of cancer cells, partially counteracting the Warburg effect and thereby limiting the metabolic flexibility required for rapid tumor proliferation.

### 3.6 Nuclear Morphological Alterations in HeLa Cells Treated With Medicinal Plant Extracts

**Figure 6:**
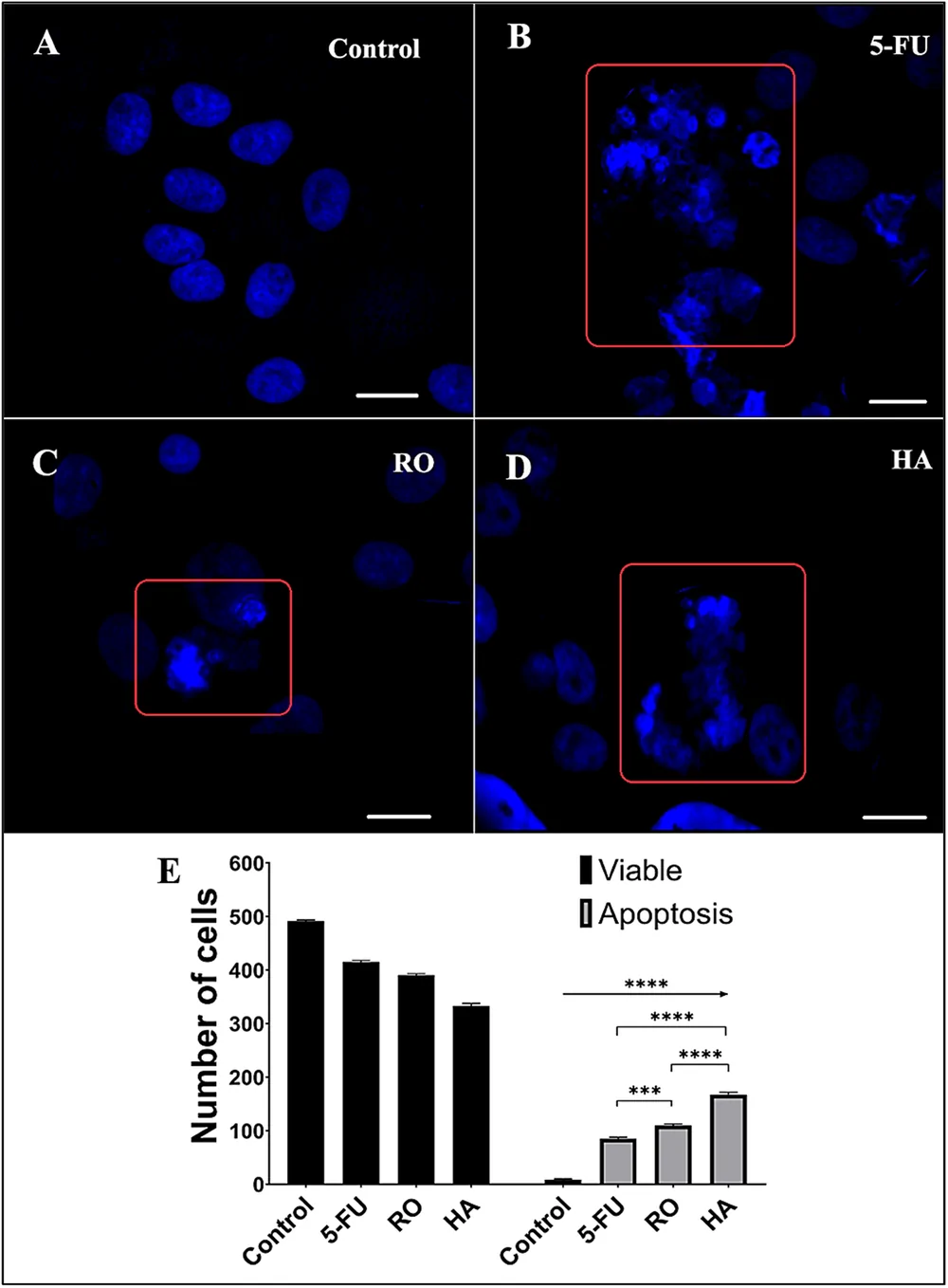
Hoechst 33258 staining (blue) to visualize apoptosis‐associated nuclear morphology. Red boxes indicate cells exhibiting apoptosis‐associated nuclear alterations. The scale bar is 100 μm. (A) Untreated control cells display intact nuclei with smooth contours and diffuse, homogeneous fluorescence, indicating normal cellular morphology. (B) 5‐FU treatment induces apoptotic changes characterized by chromatin condensation and nuclear shrinkage. (C) RO treatment increases the number of apoptotic cells exhibiting pyknotic nuclei and condensed chromatin. (D) HA treatment results in pronounced nuclear fragmentation and intense chromatin condensation, indicating advanced apoptotic morphology. (E) Quantification of viable and apoptotic cells following Hoechst 33258 staining. Treatment groups show a significant increase in apoptotic rate compared with the control. ***p < 0.001 vs control; **p < 0.01 vs control; *p < 0.05 as indicated. Data are presented as mean ± SD from three independent experiments.

Fluorescence microscopy revealed substantial treatment‐dependent alterations in nuclear morphology (Figure 6). Unlike the metabolic assays presented in Figures 2, 3, 4, 5, Figure 6 was designed to illustrate representative apoptosis‐associated nuclear alterations. Therefore, RO and HA were selected because these extracts have consistently demonstrated pronounced cytotoxic, pro‐oxidant, and apoptosis‐related activity in our previous investigations [6, 15, 20, 21], and in the present study, they produced the clearest chromatin condensation and nuclear fragmentation detectable by Hoechst staining. In contrast, IH and AS were characterized predominantly by their metabolic effects, which are reflected in the biochemical analyzes rather than in representative nuclear morphology images. Hoechst 33258 staining was employed as a morphological assay to visualize chromatin condensation, nuclear shrinkage, and fragmentation, which are widely recognized apoptosis‐associated nuclear features. These observations provide morphological evidence consistent with apoptosis; however, they should not be interpreted as definitive discrimination between apoptosis and other forms of cell death in the absence of complementary quantitative assays [23]. Control cells displayed intact, uniformly stained nuclei, consistent with normal cellular viability. In contrast, cells treated with 5‐FU, RO, and HA exhibited pronounced alterations of nuclear morphology consistent with apoptotic features, including chromatin condensation, nuclear fragmentation, and apoptotic body formation. Quantitative analysis demonstrated a significant decrease in the proportion of cells with normal nuclear morphology, accompanied by a marked increase in cells displaying apoptosis‐like nuclear alterations compared with control (***p < 0.001). Among the tested treatments, HA induced the most pronounced apoptosis‐associated nuclear alterations, showing the lowest proportion of morphologically normal cells and the highest proportion of cells exhibiting chromatin condensation and fragmentation, whereas 5‐FU and RO produced moderate but significant effects. Collectively, these findings indicate that the tested treatments suppress cell viability and induce apoptosis‐associated nuclear morphological alterations, with HA showing the strongest cytotoxic effect under the experimental conditions.

## 4 Discussion

### 4.1 Natural Compounds as Potential Anticancer Agents

Finding novel treatments that are as effective as or safer than conventional chemotherapeutics remains a major challenge. Natural compounds, including plant extracts, have garnered attention for their ability to induce apoptosis and modulate key cancer‐related pathways [13, 23, 28]. In our study, three plant extracts—IH, HA, and AS**—**were tested individually and in combination with pharmacological inhibitors of l‐arginine metabolism. Our results demonstrated that these extracts, alone or in combination with l‐NAME and nor‐NOHA, reduced HeLa cell viability, particularly at 72 h. Notably, IH exhibited the strongest cytotoxic effect, consistent with previous reports indicating its pro‐apoptotic properties [29]. These findings support the notion that plant‐derived compounds can complement existing therapies by targeting multiple mechanisms involved in tumor progression. Interestingly, when plant extracts were combined with l‐NAME, an NOS inhibitor, cytotoxicity was more pronounced than in combinations including nor‐NOHA, an arginase inhibitor. This suggests that inhibition of NOS redirects l‐arginine metabolism toward the arginase pathway [30]. Under normal conditions, arginase converts l‐arginine to ornithine and urea, supporting polyamine synthesis and cell proliferation. However, in the presence of plant extracts, arginase activity is partially suppressed, limiting polyamine production and thereby enhancing cell death. In contrast, nor‐NOHA preserves arginine for NO synthesis, which, under certain redox conditions, can facilitate adaptive responses and reduce cytotoxicity [15]. These findings are consistent with the observed increases in nitrite levels and NOS activity under plant treatments, highlighting the complex interplay between arginine metabolism, oxidative signaling, and the cytotoxic effects of natural compounds [15, 31]. The previously reported HPLC–MS/MS analysis of the investigated plant species revealed the presence of several phenolic compounds known for their antioxidant and pro‐apoptotic activities [6, 20, 22]. These molecules may contribute to the observed increase in oxidative stress markers and alterations in cellular metabolism observed in the present study.

Although normal cells were not included in the present study, our previous work demonstrated that the investigated extracts exhibited limited toxicity toward normal human retinal endothelial cells (HREC) while maintaining anticancer activity against MDA‐MB‐231 and MCF‐7 breast cancer cells, suggesting a degree of cancer‐cell selectivity [22]. These findings provide preliminary evidence supporting a favorable therapeutic window; however, the selectivity of these extracts in HeLa versus normal cervical epithelial cells remains to be established and warrants further investigation.

An important observation emerging from both the present study and our previous investigations is that these Armenian medicinal plants should not be regarded as functionally interchangeable anticancer agents. Instead, each extract appears to possess a distinct pharmacological profile. *Hypericum alpestre* primarily induces oxidative stress‐associated cytotoxicity and apoptosis [6, 20, 21], whereas *Inula helenium* and *Alchemilla smirnovii* preferentially modulate cancer cell metabolism [22]. *Rumex obtusifolius* exhibits characteristics of both mechanisms by combining metabolic modulation with direct cytotoxic activity [15, 19]. This mechanistic diversity explains why different extracts were selected for different experimental endpoints throughout the study.

### 4.2 Modulation of l‐Arginine Metabolism

l‐arginine metabolism is critical for cancer progression and immune regulation. Arginase depletes extracellular arginine, impairing T‐cell proliferation and function. At the same time, inducible nitric oxide synthase (iNOS) converts arginine into nitric oxide (NO), a signaling molecule whose biological effects depend strongly on cellular redox conditions and concentration [31, 32]. In our study, IH and AS decreased arginase activity while increasing NOS activity, favoring NO over polyamine synthesis. HA showed weaker NOS activation but retained cytotoxicity. Combined with l‐NAME, IH cytotoxicity increased, likely due to arginine being redirected to arginase, though plant‐mediated arginase inhibition limited cell survival. Conversely, nor‐NOHA preserved arginine for NO production, resulting in lower cytotoxicity. Nitrite levels mirrored these effects, confirming NO/NOS pathway activation [33, 34]. Comparable studies have highlighted the importance of arginine metabolism in cancer. For example, NOHA‐mediated arginase inhibition reduced proliferation and induced apoptosis in arginase‐expressing breast cancer cells by limiting polyamine synthesis, while preserving arginine for NO‐mediated immune activity [32, 33]. Similarly, in murine renal carcinoma models, arginase inhibition restored T‐cell function and suppressed tumor growth [35]. Our findings align with these observations, demonstrating that targeting arginine metabolism—particularly with natural compounds—can simultaneously impair tumor‐supportive pathways and enhance antitumor immune signaling, providing a promising combinatorial strategy for cancer therapy.

### 4.3 Oxidative Stress and Antioxidant Responses

Cancer cells rely on a finely regulated balance of ROS to sustain proliferation and survival while avoiding oxidative damage. While moderate ROS levels act as signaling mediators that promote tumor growth, angiogenesis, and metabolic adaptation, excessive ROS accumulation can trigger lipid peroxidation, mitochondrial dysfunction, and apoptosis [36]. In our study, treatment with plant extracts generally increased MDA levels, indicating enhanced lipid peroxidation and oxidative stress. Concurrently, SOD and catalase activities were elevated, reflecting an adaptive antioxidant response [37]. Proline levels were also modulated, consistent with stress‐protective metabolic adaptations. Together, these findings suggest that the plant extracts induce apoptosis‐associated changes in HeLa cells by shifting the redox balance toward ROS‐mediated cytotoxicity, while the initial increase in antioxidant enzyme activity may represent a compensatory response to oxidative stress [37, 38]. Similar observations have been reported in studies with other plant‐derived compounds, where treatment increased ROS and lipid peroxidation while transiently enhancing antioxidant defenses, ultimately leading to cancer cell apoptosis [39]. Our results support these findings and extend them by showing that different plant extracts can exert distinct redox effects depending on concentration and exposure time, highlighting the nuanced role of oxidative stress modulation in natural compound‐mediated anticancer strategies.

### 4.4 Proline Level

Proline is a major structural component of collagen and plays a critical role in extracellular matrix (ECM) integrity [40]. Collagen degradation may release free proline, potentially contributing to elevated intracellular or extracellular proline levels. In this context, increased proline concentrations could reflect enhanced matrix remodeling, a process often associated with tumor progression and metastatic potential. Conversely, the observed reduction in proline levels following treatment with certain extracts may indicate decreased collagen turnover or altered proline metabolism. Lower free proline availability might suggest stabilization of the extracellular matrix, which could limit the invasive behavior of cancer cells. However, since collagen content, matrix metalloproteinases (MMPs), and hydroxyproline levels were not directly assessed in the present study, these interpretations remain speculative. Further investigation of ECM‐related markers would be necessary to clarify the relationship between proline metabolism and metastatic potential in treated HeLa cells.

### 4.5 Energy Metabolism and the Warburg Effect

Cancer cells frequently rely on aerobic glycolysis, also known as the Warburg effect, which is characterized by high glucose consumption and lactate production even in the presence of oxygen [41, 42]. This metabolic reprogramming supports rapid proliferation, angiogenesis, and immune evasion within the tumor microenvironment. In our study, treatment with IH and AS extracts significantly reduced intracellular glucose, pyruvate, and lactate levels in HeLa cells, indicating a partial reversal of the Warburg phenotype. In contrast, 5‐FU treatment did not produce significant changes in these metabolites, suggesting that conventional chemotherapy may not directly impact this metabolic pathway under the tested conditions. These findings suggest that the plant extracts may limit the energy supply necessary for rapid cancer cell proliferation and disrupt the pro‐tumorigenic microenvironment. When combined with the previously observed modulation of l‐arginine metabolism and redox balance, these metabolic effects likely contribute to the enhanced cytotoxicity observed with plant treatments, highlighting the potential of simultaneously modulating several metabolic processes in cancer cells. Similar anti‐Warburg effects have been reported with other natural compounds, where glycolytic inhibition contributed to reduced lactate production, impaired cell proliferation, and sensitization to apoptosis [43], supporting the notion that plant‐derived metabolites can complement conventional therapies by targeting cancer metabolism. Combining plant extracts with l‐NAME and nor‐NOHA produced stronger effects on cell viability and redox balance than individual treatments, supporting previous reports that targeting the arginase and NOS pathways can suppress tumor progression and angiogenesis. Notably, some extracts, particularly *Alchemilla smirnovii*, exhibited anticancer activity comparable to that of 5‐FU, indicating intrinsic cytotoxic and metabolic‐modulatory properties.

### 4.6 Possible Signaling Mechanisms Underlying the Observed Metabolic Alterations

Although the present study focused on biochemical and metabolic endpoints, the observed changes in nitric oxide metabolism, oxidative stress, glycolysis, and proline metabolism are unlikely to occur independently. Instead, they probably reflect coordinated cellular adaptation mediated through interconnected upstream signaling pathways and their downstream metabolic and apoptotic consequences. With respect to glycolytic suppression, the reduction in glucose consumption and lactate/pyruvate accumulation observed following extract treatment is consistent with inhibition of the PI3K/Akt/mTOR axis, a central upstream regulator of glycolytic flux that promotes glucose transporter (GLUT1) trafficking and stabilizes hypoxia‐inducible factor‐1α (HIF‐1α), the principal transcriptional driver of glycolytic enzymes such as hexokinase‐2 and pyruvate dehydrogenase kinase‐1 [19, 20, 44, 45, 46, 47]. A parallel or alternative explanation involves activation of AMP‐activated protein kinase (AMPK), which senses energy stress and, once activated, suppresses mTORC1 signaling while promoting catabolic, energy‐conserving metabolic programs; AMPK activation would therefore be expected to produce a metabolic phenotype similar to that observed here [48]. Downstream of this axis, reduced glycolytic capacity is known to sensitize cancer cells to apoptosis by limiting ATP availability and NADPH‐dependent antioxidant defenses, providing a plausible mechanistic link between the metabolic changes and the reduced cell viability reported in this study [49]. Regarding the redox‐related findings (elevated SOD and catalase activity, increased MDA), these changes are compatible with activation of the NRF2‐Keap1 antioxidant response pathway, an upstream sensor of oxidative stress that transcriptionally upregulates antioxidant and detoxifying enzymes as a compensatory mechanism; sustained or excessive activation of this pathway, once cellular antioxidant capacity is overwhelmed, converges downstream on p53 stabilization and mitochondrial‐mediated apoptotic signaling, consistent with the nuclear morphology changes observed by Hoechst staining [50, 51]. Similarly, the shifts in arginase and nitric oxide synthase activity may reflect upstream regulation of arginine availability, which is sensed by mTORC1 through Rag GTPase‐dependent mechanisms and, downstream, determines the balance between polyamine biosynthesis (via ornithine decarboxylase) and nitric oxide/reactive nitrogen species production—both of which influence mitochondrial function, ROS generation, and cellular bioenergetics [5, 6, 35]. While these signaling pathways were not directly investigated in the present work—for instance, through Western blotting of phosphorylated Akt, AMPK, or HIF‐1α, or through pharmacological pathway inhibition/activation studies—they provide biologically plausible mechanistic links connecting the observed upstream regulatory changes to their downstream metabolic and apoptotic consequences. Future studies integrating metabolic profiling with targeted molecular pathway analyzes, including gene and protein expression of the candidate regulators discussed above, will be required to establish causality and to determine which of these pathways predominantly mediates the extract‐induced phenotype in HeLa cells.

### 4.7 Limitations of the Study

Despite these promising findings, several limitations of the present study should be acknowledged. First, the experiments were performed in a single cancer cell line (HeLa); therefore, validation in additional cancer models and in vivo systems is required to establish the broader anticancer relevance of the investigated extracts. Although Hoechst 33258 staining revealed chromatin condensation and other apoptosis‐associated nuclear alterations, these findings do not provide definitive confirmation of apoptosis. Our previous study showed concordant Hoechst and caspase‐3 responses; however, apoptosis was not independently confirmed here by Annexin V/PI flow cytometry or other complementary assays. Future studies should therefore combine morphological assessment with quantitative apoptosis‐specific analyzes. Furthermore, although coordinated changes in l‐arginine metabolism, oxidative stress, and cellular energy metabolism were demonstrated, the upstream signaling mechanisms were not directly investigated. Pathways such as PI3K/Akt/mTOR, AMPK, HIF‐1α, NRF2, and p53 may contribute to these responses, but their involvement remains to be experimentally established. Finally, the present study did not directly assess selectivity toward non‐malignant cervical cells. Nevertheless, our recent study showed that IH, AS, and RO exhibited limited cytotoxicity toward normal human retinal endothelial cells (HREC) while retaining activity against MDA‐MB‐231 and MCF‐7 cells [22], providing preliminary evidence of preferential activity toward malignant cells. However, these findings cannot be directly extrapolated to HeLa cells. Further studies should therefore evaluate normal cervical cells and assess the long‐term efficacy and safety of the most promising extract–inhibitor combinations in appropriate preclinical models. Overall, these limitations indicate that the present findings should be considered supportive mechanistic and phenotypic evidence rather than definitive characterization of the underlying molecular mechanisms or therapeutic selectivity.

## 5 Conclusion

**Figure 7:**
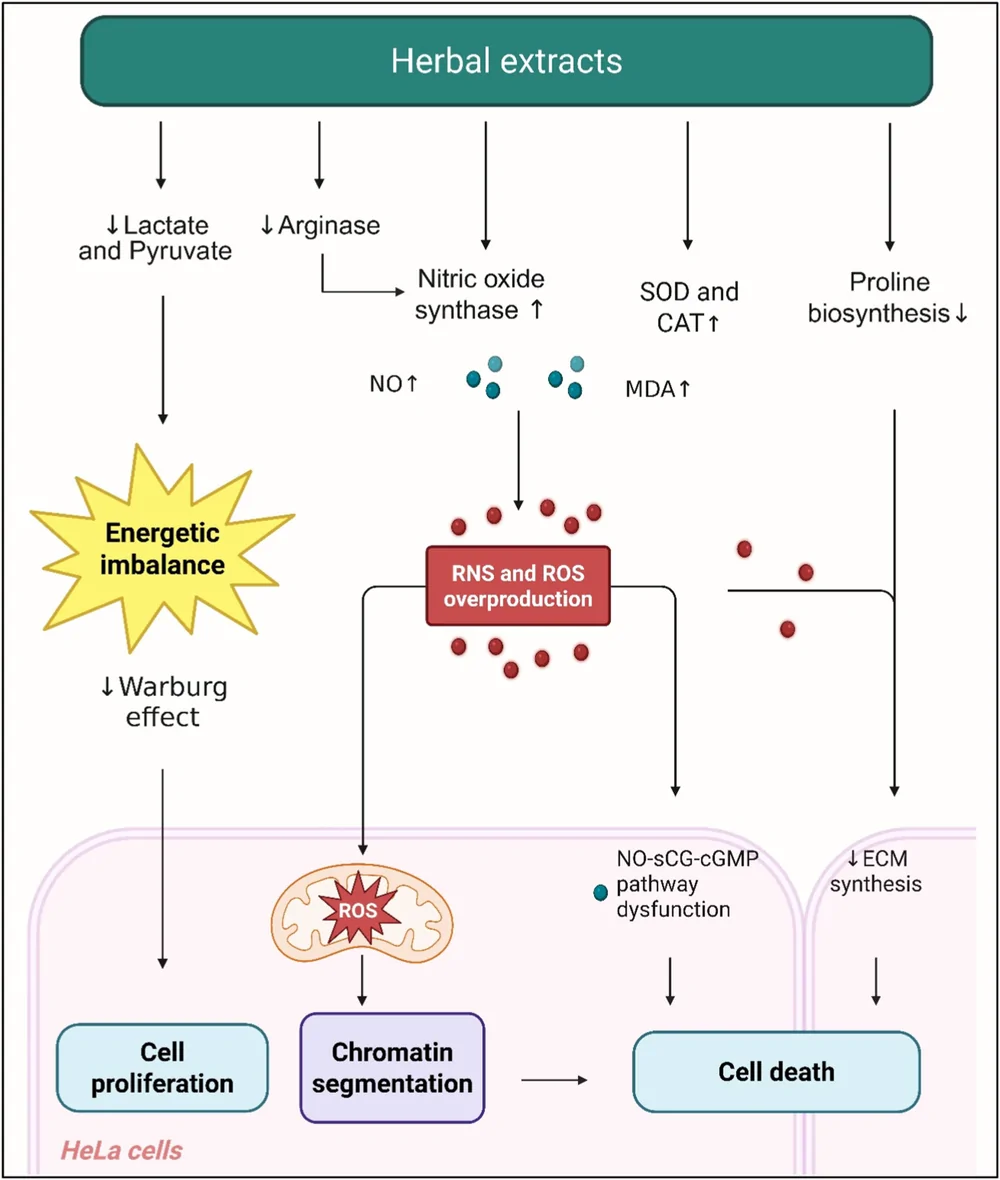
The schematic summarizes the proposed mechanism by which herbal extracts exert cytotoxic effects in HeLa cells through oxidative and nitrosative stress–mediated pathways. Treatment with herbal extracts reduces arginase activity, thereby increasing nitric oxide synthase activity and nitric oxide (NO) production. Simultaneously, the extracts modulate antioxidant defense systems, influencing superoxide dismutase (SOD) and catalase (CAT) activities, and also affect malondialdehyde (MDA) levels, indicating alterations in lipid peroxidation and oxidative stress. In addition, the extracts reduce proline biosynthesis, suggesting changes in amino acid metabolism that may influence extracellular matrix dynamics. The combined metabolic and redox alterations contribute to an overproduction of ROS and reactive nitrogen species (RNS) within the cells. The excessive accumulation of ROS and RNS disrupts several critical cellular pathways. Elevated mitochondrial oxidative stress promotes chromatin segmentation, while dysregulation of the NO–sGC–cGMP signaling pathway and suppression of extracellular matrix (ECM) synthesis further impair cellular integrity and survival. Figure was created using BioRender.com.

Collectively, these findings demonstrate that IH, HA, RO, and AS extracts exert cytotoxic effects in HeLa cells through distinct but interconnected mechanisms, involving modulation of l‐arginine and proline metabolism, glycolytic activity, and redox homeostasis (Figure 7). IH and AS predominantly affected cellular energy metabolism, evidenced by reduced glucose, pyruvate, and lactate levels, with AS producing metabolic and redox alterations in some aspects comparable to or exceeding those observed with 5‐FU under the tested conditions. In contrast, HA and RO were associated with the most pronounced apoptosis‐associated nuclear morphology changes, including chromatin condensation and nuclear fragmentation, consistent with their previously reported pro‐apoptotic activity. Combination with l‐arginine metabolism inhibitors further enhanced several of these effects, supporting the potential of these extracts as complementary strategies alongside conventional chemotherapy. Rather than acting through a single molecular target, the tested extracts appear to engage complementary metabolic and redox vulnerabilities of cancer cells—some primarily through energetic reprogramming, others through direct induction of apoptotic morphology. Further studies, including apoptosis‐specific assays (e.g., Annexin V/PI flow cytometry), molecular signaling analyzes, in vivo validation, and evaluation in non‐malignant cervical cells, are required to define the precise mechanisms, therapeutic selectivity, and long‐term efficacy of each extract.

## Author Contributions

**Mery Kocharyan:** investigation, methodology, software, formal analysis. **Gohar Sevoyan:** investigation, methodology, software, formal analysis. **Hasmik Karapetyan:** investigation, methodology, software, formal analysis. **Anzhela Sargsyan:** investigation, methodology, software, formal analysis. **Mikayel Ginovyan:** conceptualization, methodology, data curation, writing – original draft, writing – review and editing. **Nikolay Avtandilyan:** conceptualization, methodology, writing – review and editing, funding acquisition, resources. **Hayarpi Javrushyan:** conceptualization, methodology, writing – review and editing, funding acquisition, supervision.

## Conflicts of Interest

The authors declare no conflicts of interest.

## Supporting information


Supporting File 1

Supporting File 2

## Data Availability

The data that support the findings of this study are available from the corresponding author upon reasonable request.
