# Supplementary material for: Cytotoxic Effects of Medicinal Plant Extracts on HeLa Cells via Redox Imbalance, L‐Arginine Metabolic Reprogramming, and Glycolytic Suppression
Source: Cell Biochem Funct. 2026 Sep 11;44(9):e70307. doi: 10.1002/cbf.70307 (PMC13566611; doi:10.1002/cbf.70307)
Supplement: Supplementary file 1 [file CBF-44-e70307-s001.docx]

**Methods: LC-Q-Orbitrap HRMS analysis of *Rumex obtusifolius, Alchemilla smirnovii* Juz, and *Inula helenium* extracts**

1.1. **LC-Q-Orbitrap HRMS analysis of plant extracts**

Qualitative analyses were performed on a Dionex UltiMate 3000 UHPLC system (Thermo Fisher Scientific, San Jose, CA, USA). For AS and IH*,* a Kinetex® column (150 × 4.6 mm, 5 μm; Phenomenex) was used, whereas for RO, separations were performed on a Synergi™ Hydro-RP A column (150 × 4.5 mm, 4 μm; Phenomenex). The mobile phase consisted of water (A) and acetonitrile (B), each containing 0.1% (v/v) formic acid; flow rate 0.8 mL min⁻¹. Injection volumes and gradients were as follows: *Alchemilla smirnovii* Juz.: 2 μL; 0 min, 10% B; 25 min, 25% B; 27 min, 100% B; 30 min, 100% B; *Inula helenium*: 6 μL; 0 min, 10% B; 20 min, 50% B; 25 min, 100% B; 30 min, 100% B; *Rumex obtusifolius*: 1 μL; 0 min, 10% B; 25 min, 40% B; 30 min, 100% B; 35 min, 100% B.

The chromatographic system was coupled to a PDA detector (UV–Vis) and a Q Exactive™ Focus quadrupole-Orbitrap mass spectrometer (Thermo Fisher Scientific, Bremen, Germany) equipped with a HESI-II source. MS detection was carried out in negative ion mode with the following source settings: sheath/aux/sweep gas 35/15/3 (arb), spray voltage 2.5 kV, S-lens RF 50, capillary 350 °C, heater 300 °C. Full-scan MS: resolution 70,000 (at *m/z* 200), AGC target 1.0 × 10⁶, maximum injection time auto, scan range *m/z* 120–1200. Data-dependent MS²: resolution 17,500, isolation window 3.0 *m/z*, normalized collision energy (NCE) 30, AGC target 1.0 × 10⁶, maximum injection time auto.

Raw high-resolution MS data were processed in Compound Discoverer (v. 2.1; Thermo Fisher Scientific, Waltham, MA, USA). Major metabolites were annotated based on accurate mass and MS/MS fragmentation patterns by matching against a custom in-house library of phytochemicals compiled from the literature and implemented within the software.

1.2. **Semi-quantitative analysis of major phenolic compounds by LC-PDA**

Standard solutions were prepared by dissolving 1 mg of each reference compound in 1 mL of 70% (v/v) ethanol. Exception: ellagic acid was first dissolved as a 1 mg mL⁻¹ stock in 0.1 M NaOH. Calibration curves were obtained by LC–PDA analysis of serial dilutions of the standards prepared in 70% ethanol, integrating peak areas at 270 nm (e.g., flavan-3-ols, ellagitannins/ellagic acid, flavonols) or 325 nm (hydroxycinnamic acids). Limits of detection (LOD) and quantification (LOQ) were defined as the absolute amounts giving signal-to-noise ratios of 3:1 and 10:1, respectively, using peak height. Noise was taken as the baseline fluctuation in blank injections (70% ethanol) measured in a retention-time window adjacent to each analyte peak. Linear regression (peak area vs. concentration) was used to obtain the calibration equation and coefficient of determination (r²). For analytes with available standards, contents were determined by external calibration with the corresponding authentic standard. For analytes without available standards, contents were semi-quantified against a structurally related compound from the same class, and are reported as equivalents (e.g., ellagitannins as ellagic acid equivalents, flavan-3-ols as (+)-catechin equivalents, hydroxycinnamates as chlorogenic acid equivalents). The chromatographic column and separation conditions were identical to those used for the LC-Q-Orbitrap HRMS method [1,2].

References

[1]	B. Kusznierewicz, A. Piasek, A. Bartoszek, J. Namiesnik, The optimisation of analytical parameters for routine profiling of antioxidants in complex mixtures by HPLC coupled post-column derivatisation, Phytochemical Analysis 22 (2011) 392–402. https://doi.org/10.1002/pca.1294.

[2]	B. Kusznierewicz, A. Piasek, A. Bartoszek, J. Namiesnik, Application of a commercially available derivatization instrument and commonly used reagents to HPLC on-line determination of antioxidants, Journal of Food Composition and Analysis 24 (2011) 1073–1080. https://doi.org/10.1016/j.jfca.2011.01.010.

**Sup.table 1.** Retention time (RT, min), proposed formula, theoretical *m/z* value of the precursor ion (Da), experimental *m/z* value of the precursor ion (Da), accuracy (Δm, ppm), *m/z* values and formulae of diagnostic fragment ions (Da), and content (mg/g DW) of major compounds identified in *Alchemilla* *smirnovii* Juz. extracts.

| **No** | **RT [min]** | **Tentative Identification** | **Formula** | **Theoretical**  **[M − H]^−^** | **Experimental**  **[M − H]^−^** | **Δm [ppm]** | **Product ions** | | **Content (mg/g DW)** |
| --- | --- | --- | --- | --- | --- | --- | --- | --- | --- |
|  |  |  |  |  |  |  | **Neutral-loss** | ***m/z* (formula)** |  |
| **1** | 4.43 | Procyanidin dimer | C_30_H_26_O_12_ | 577.13460 | 577.13482 | -0.38 | [M − H − (epi)Cat]^−^  [M − H − C_8_H_8_O_3_ − H_2_O]^−^  [M − H − (epi)Cat − C_9_H_8_O_3_]^−^  [M − H − (epi)Cat − C_6_H_8_O_3_]^−^  [M − H − (epi)Cat − CO_2_]^−^  [M − H − C_8_H_8_O_3_]^−^ | 289.07169 (C_15_H_13_O_6_)  407.07706 (C_22_H_15_O_8_)  125.02310 (C_6_H_5_O_3_) 161.02336 (C_9_H_5_O_3_)  245.08173 (C_14_H_13_O_4_)  425.08719 (C_22_H_17_O_9_) | 0.333 ± 0.029^(b)^ |
| **2** | 4.82 | (+)-Catechin | C_15_H_14_O_6_ | 289.07121 | 289.07135 | -0.48 | [M − H − C_9_H_8_O_4_]^−^  [M − H − C_8_H_6_O_4_]^−^  [M − H − C_9_H_8_O_3_]^−^  [M − H − C_7_H_6_O_3_]^−^  [M − H − C_8_H_8_O_3_]^−^  [M − H − C_2_H_2_O − CO_2_]^−^ | 109.02821 (C_6_H_5_O_2_)  123.04388 (C_7_H_7_O_2_)  125.02319 (C_6_H_5_O_3_)  151.03902 (C_8_H_7_O_3_)  137.02324 (C_7_H_5_O_3_)  203.07104 (C_12_H_11_O_3_) | 0.384 ± 0.049^(a)^ |
| **3** | 5.04 | Caffeoylquinic acid | C_16_H_18_O_9_ | 353.08726 | 353.08742 | -0.47 | [M − H − caffeoyl]^−^  [M − H − quinoyl − H_2_O]^−^  [M − H − caffeoyl − H_2_O]^−^  [M − H − quinoyl − CO_2_]^−^ | 191.05531 (C_7_H_11_O_6_)  161.02324 (C_9_H_5_O_3_)  173.04398 (C_7_H_9_O_5_)  135.04356 (C_8_H_7_O_2_) | 0.376 ± 0.063^(a)^ |
| **4** | 5.42 | Brevifolincarboxylic acid | C_13_H_8_O_8_ | 291.01409 | 291.01427 | -0.62 | [M − H − CO_2_]^−^  [M − H − CO_2_ − 2CO]^−^  [M − H − CO_2_ − CO]^−^  [M − H − 2CO_2_ − 2CO]^−^  [M − H − CO_2_ − 2CO − H_2_O]^−^ | 247.02458 (C_12_H_7_O_6_)  191.03412 (C_10_H_7_O_4_)  219.02928 (C_11_H_7_O_5_)  147.04416 (C_9_H_7_O_2_)  173.02365 (C_10_H_5_O_3_) | 0.780 ± 0.058^(c)^ |
| **5** | 7.37 | Myricetin-glucuronide | C_21_H_18_O_14_ | 493.06183 | 493.06215 | -0.65 | [M − H − HA]^−^  [M − H − HA − H_2_O]^−^ | 317.0304 (C_15_H_9_O_8_)  299.0199 (C_15_H_7_O_7_) | 0.802 ± 0.034^(d)^ |
| **6** | 7.81 | Casuarinin (I) | C_41_H_28_O_26_ | 935.07906 | 935.07933 | -0.29 | [(M − H) − galloyl − HHDP − Hex − H_2_O]^−^  [(M − H) − 2HHDP − Hex]^−^  [M − H − C_28_H_20_O_19_]^−^  [M − H − C_29_H_18_O_20_]^−^  [M − H − C_28_H_22_O_19_]^−^ | 300.99905 (C_14_H_5_O_8_)  169.01337(C_7_H_5_O_5_)  275.01984 (C_13_H_7_O_7_)  249.04041 (C_12_H_9_O_6_)  273.0043 (C_13_H_5_O_7_) | 0.525 ± 0.021^(c)^ |
| **7** | 8.35 | Ferulic acid sulfate | C_10_H_10_O_7_S | 273.00690 | 273.00716 | -0.97 | [M − H − SO_3_ − CO_2_ − CH_3_]^−^  [M − H − SO_3_ − CH_3_]^−^  (M − H − SO_3_ − CO_2_]^−^  [M − H − SO_3_]^−^ | 134.03615 (C_8_H_6_O_2_)  178.02629 (C_9_H_6_O_4_)  149.05972 (C_9_H_9_O_2_)  193.04991 (C_10_H_9_O_4_) | 0.181 ± 0.016^(e)^ |
| **8** | 8.91 | Casuarinin (II) | C_41_H_28_O_26_ | 935.07906 | 936.08716 | -0.29 | [M − H − galloyl − HHDP − Hex − H_2_O]^−^  [M − H − 2HHDP − Hex]^−^  [M − H − C_28_H_20_O_19_]^−^  [M − H − C_29_H_18_O_20_]^−^  [M − H − C_28_H_22_O_19_]^−^ | 300.99908 (C_14_H_5_O_8_)  169.01335 (C_7_H_5_O_5_)  275.01981(C_13_H_7_O_7_)  249.04031 (C_12_H_9_O_6_)  273.00528 (C_13_H_5_O_7_) | 0.431 ± 0.014^(c)^ |
| **9** | 9.52 | *p*-Coumaroylquinic acid | C_16_H_18_O_8_ | 337.09234 | 337.09258 | -0.71 | [M − H − coumaroyl]^−^  [M − H − quinoyl − CO_2_]^−^  [M − H − quinoyl]^−^ | 191.05537 (C_7_H_11_O_6_)  119.04896 (C_8_H_7_O)  163.03877 (C_9_H_7_O_3_) | 0.243 ± 0.049^(f)^ |
| **10** | 10.79 | Quercetin-glucuronide-rhamnoside | C_27_H_28_O_17_ | 623.12483 | 623.12516 | -0.54 | [M − H − HA − Rha]^−^  [M − H − C_15_H_10_O_7_]^−^  [M − H − HA]^−^ | 301.03546 (C_15_H_9_O_7_)  321.08264 (C_12_H_17_O_10_)  447.09244 (C_21_H_19_O_11_) | 0.0932 ± 0.0065^(d)^ |
| **11** | 10.92 | Ellagic acid-*O*-xyloside | C_19_H_14_O_12_ | 433.040705 | 433.04094 | -0.55 | [(M − H) − Pent]^−^ | 300.9985 (C_14_H_5_O_8_) | 1.00  ± 0.12^(c)^ |
| **12** | 12.09 | Ellagic acid | C_14_H_6_O_8_ | 300.99844 | 300.99859 | -0.50 | [(M − H) − CO − CO_2_]^−^  [(M − H) − H_2_O]^−^  [(M − H) − CO − 2CO_2_]^−^  [(M − H) − 2CO − CO_2_]^−^  [(M − H) − CO_2_]^−^  [(M − H) − 2CO]^−^  [(M − H) − 3CO − CO_2_]^−^ | 229.01389 (C_12_H_5_O_5_)  283.9964 (C_14_H_4_O_7_)  185.02371 (C_11_H_5_O_3_)  201.01884 (C_11_H_5_O_4)_  257.00891 (C_13_H_5_O_6_)  245.00908 (C_12_H_5_O_6_)  173.02377 (C_10_H_5_O_3_) | 2.58  ± 0.23^(a)^ |
| **13** | 12.77 | Rutin | C_27_H_30_O_16_ | 609.14556 | 609.14580 | -0.39 | [M − H − Rha − Hex]^−^ | 301.03503 (C_15_H_9_O_7_) | 0.193  ± 0.014^(d)^ |
| **14** | 12.95 | Agrimoniin | C_82_H_54_O_52_ | 1869.15030 | 1869.15204  (934.073  [M − 2H]^2−^) | -0.92 | [M − H − Pot − HHDP − galloyl − Glu]^−^  [M − H − Pot]^−^  [M − H − Pot − galloyl]^−^  [M − H − Pot − HHDP]^−^ | 300.99905 (C_14_H_5_O_8_)  935.07843 (C_41_H_27_O_26_)  783.06964 (C_34_H_23_O_22_)  633.07239 (C_27_H_21_O_18_) | 5.71  ± 0.43^(c)^ |
| **15** | 13.17 | Quercetin-*O*-glucuronide | C_21_H_18_O_13_ | 477.06692 | 477.06703 | -0.24 | [M − H − HA]^−^ | 301.03543 (C_15_H_9_O_7_) | 2.100 ± 0.048^(d)^ |
| **16** | 16.71 | Ducheside A | C_20_H_16_O_12_ | 447.05635 | 447.05682 | -1.05 | [M − H −Pent]^−^  [M − H − Pent − CH_3_]^−•^ | 315.01459 (C_15_H_7_O_8_)  299.99106 (C_14_H_4_O_8_) | 0.702 ± 0.023^(c)^ |

Qualitative analysis was performed using LC-Q-Orbitrap HRMS in negative ion mode. The abbreviations used are as follows: caffeoyl, −C_9_H_6_O_3_, *m/z* 162.031; (epi)Cat (epicatechin/catechin), −C_15_H_12_O_6_, *m/z* 288.063; galloyl, −C_7_H_4_O_4_, *m/z* 152.011; Glu (glucose), C_6_H_12_O_6_, *m/z* 180.063; HA (hexuronic acid), −C_6_H_8_O_6_, *m/z* 176.032; Hex (hexose), −C_6_H_10_O_5_, *m/z* 162.053; HHDP (hexahydroxydiphenoyl), −C_14_H_6_O_8_, *m/z* 302.006; Pent (pentose), −C_5_H_8_O_4_, *m/z* 132.042; Pot (potentillin), −C_41_H_26_O_26_, *m/z* 934.071; Rha (rhamnose), −C_6_H_10_O_4_, *m/z* 146.058 quinoyl, −C_7_H_10_O_5_, *m/z* 174.052. Quantitative analysis was performed based on LC-PAD analyses and the content of individual analytes was calculated based: (a) on authentic standards; (b) as catechin equivalents; (c) as ellagic acid equivalents; (d) as quercetin equivalents; (e) as ferulic acid equivalents (f) as chlorogenic acid equivalents. The data are expressed as mean values of three independent experiments ± standard deviation. Basic validation parameters (external calibration; linear regression): (+)-catechin: linear range 10–40 µg mL⁻¹; calibration curve *y* = 1.829*x* − 3.429; *r*² = 0.997; LOD 4.92 µg mL⁻¹; LOQ 16.41 µg mL⁻¹, ellagic acid: linear range 50–500 µg mL⁻¹; calibration curve *y* = 4.366*x* − 72.710; *r*² = 0.992; LOD 2.06 µg mL⁻¹; LOQ 6.87 µg mL⁻¹, chlorogenic acid: linear range 25–400 µg mL⁻¹; calibration curve *y* = 6.646*x* − 2.009; *r*² = 0.999; LOD 1.35 µg mL⁻¹; LOQ 4.51 µg mL⁻¹, quercetin: linear range 10–125 µg mL⁻¹; calibration curve *y* = 7.716*x* − 18.615; *r*² = 0.998; LOD 1.17 µg mL⁻¹; LOQ 3.89 µg mL⁻¹, ferulic acid: linear range 25–150 µg mL⁻¹; calibration curve *y* = 4.698*x* − 5.267; *r*² = 0.998; LOD 0.09 µg mL⁻¹; LOQ 0.29 µg mL⁻¹. *Notes:* *x* — analyte concentration (µg mL⁻¹); *y* — peak area at the detection wavelength.

**Sup.table 2.** Retention time (RT, min), proposed formula, theoretical *m/z* value of the precursor ion (Da), experimental *m/z* value of the precursor ion (Da), accuracy (Δm, ppm), *m/z* values and formulae of diagnostic fragment ions (Da), and content (mg/g DW) of major compounds identified in *Inula Helenium* extracts.

| **No** | **RT [min]** | **Tentative Identification** | **Formula** | **Theoretical**  **[M − H]^−^** | **Experimental**  **[M − H]^−^** | **Δm [ppm]** | **Product ions** | | **Content (mg/g DW)** |
| --- | --- | --- | --- | --- | --- | --- | --- | --- | --- |
|  |  |  |  |  |  |  | **Neutral-loss** | ***m/z* (formula)** |  |
| **1** | 3.67 | Caffeoylquinic acid (I) | C_16_H_18_O_9_ | 353.08726 | 353.08737 | -0.32 | [M − H − caffeoyl]^−^  [M − H − quinoyl − H_2_O]^−^  [M − H − caffeoyl − H_2_O]^−^  [M − H − quinoyl − CO_2_]^−^  [M − H − quinoyl]^−^ | 191.05536 (C_7_H_11_O_6_) 161.02325 (C_9_H_5_O_3_) 173.04469 (C_7_H_9_O_5_) 135.04385 (C_8_H_7_O_2_) 179.03442 (C_9_H_7_O_4_) | 0.0106  ±0.0059^(b)^ |
| **2** | 4.81 | Caffeoylquinic acid (II) | C_16_H_18_O_9_ | 353.08726 | 353.08735 | -0.27 | [M − H − caffeoyl]^−^  [M − H − quinoyl − CO_2_]^−^  [M − H − quinoyl]^−^ | 191.05536 (C_7_H_11_O_6_)  135.04395 (C_8_H_7_O_2_)  179.03412 (C_9_H_7_O_4_) | 0.12204  ±0.00046^(a)^ |
| **3** | 8.22 | Quercetin-*O*-hexoside | C_21_H_20_O_12_ | 463.08765 | 463.08798 | -0.71 | [M − H − Hex]^−^ | 301.0343 (C_15_H_9_O_7_) | 0.14595  ± 0.00063^(d)^ |
| **4** | 8.58 | Pentahydroxy-monomethoxyflavone hexoside | C_22_H_22_O_13_ | 493.09822 | 493.09873 | -1.04 | [M − H − Hex]^−^  [M − H− Hex − CH_3_]^−•^ | 331.04474 (C_16_H_11_O_8_) 316.02219 (C_15_H_8_O_8_) | 0.12725 ±0.00092^(d)^ |
| **5** | 9.15 | Dicaffeoylquinic acid (I) | C_25_H_24_O_12_ | 515.11895 | 515.11914 | -0.38 | [M − H − 2caffeoyl − H_2_O]^−^  [M − H − caffeoyl − quinoyl]^−^  [M − H − 2caffeoyl]^−^  [M − H − caffeoyl]^−^  [M − H − quinoyl − caffeoyl − CO_2_]^−^ | 173.04462 (C_7_H_9_O_5_) 179.03407 (C_9_H_7_O_4_) 191.05531(C_7_H_11_O_6_) 353.08795 (C_16_H_17_O_9_) 135.04411 (C_8_H_7_O_2_) | 1.354  ±0.064^(b)^ |
| **6** | 9.52 | Caffeoyl hexoside derivative | C_26_H_32_O_12_ | 535.18155 | 535.18182 | -0.50 | [M − H − C_11_H_14_O_3_ − H_2_O]^−^  [M − H − C_11_H_14_O_3_ − Hex − H_2_O]^−^  [M − H − C_11_H_14_O_3_ − Hex]^−^  [M − H − caffeoyl − Hex]^−^ | 323.07724 (C_15_H_15_O_8_) 161.02338 (C_9_H_5_O_3_) 179.03403 (C_9_H_7_O_4_)  211.09698 (C_11_H_15_O_4_) | 0.071  ±0.010^(c)^ |
| **7** | 9.96 | Dicaffeoylquinic acid (II) | C_25_H_24_O_12_ | 515.11895 | 515.11914 | -0.38 | [M − H − quinoyl − caffeoyl − CO_2_]^−^ | 135.04399 (C_8_H_7_O_2_) | 0.094  ±0.016^(b)^ |
| **8** | 10.19 | Kaempferol-*O*-maloylhexoside | C_24_H_22_O_14_ | 533.09313 | 533.09356 | -0.81 | [M − H − malonyl − Hex]^−^ | 285.03998 (C_15_H_9_O_6_) | 0.02944  ±0.00019^(e)^ |
| **9** | 12.57 | Quercetin | C_15_H_10_O_7_ | 301.03483 | 301.03493 | -0.35 | [M − H − C_8_H_6_O_3_]^−^  [M − H − C_8_H_4_O_5_]^−^ | 151.00262 (C_7_H_3_O_4_) 121.02824 (C_7_H_5_O_2_) | 0.1579 ±0.0010^(a)^ |
| **10** | 12.94 | Tricaffeyolquinic acid | C_34_H_30_O_15_ | 677.150650 | 677.15137 | -1.07 | [M − H − caffeoyl]^−^  [M − H − 2caffeoyl]^−^ | 515.11969 (C_25_H_23_O_12_)  353.08798 (C_16_H_17_O_9_) | 0.00927 ±0.00076^(b)^ |
| **11** | 13.25 | Caffeoyl hexoside derivative | C_29_H_30_O_13_ | 585.16082 | 585.16138 | -0.96 | [M − H − Hex − C_14_H_12_O_4_]^−^  [M − H − Hex − caffeoyl − C_3_H_6_O]^−^  [M − H − caffeoyl − C_4_H_8_O_2_]^−^  [M − H − caffeoyl]^−^  [M − H − Hex − C_14_H_12_O_4_ − H_2_O]^−^  [M − H − caffeoyl − C_4_H_8_O_2_ − 2H_2_O]^−^ | 179.03412 (C_9_H_7_O_4_)  203.03444 (C_11_H_7_O_4_)  335.07751 (C_16_H_15_O_8_) 423.12967 (C_20_H_23_O_10_) 161.02342 (C_9_H_5_O_3_) 299.05646 (C_16_H_11_O_6_) | 0.00794 ±0.00072^(c)^ |
| **12** | 13.59 | Isorhamnetin | C_16_H_12_O_7_ | 315.05048 | 315.05072 | -0.78 | [(M − H) − CH_3_]^−•^  [M − H − C_9_H_8_O_3_]^−^ | 300.02762 (C_15_H_8_O_7_) 151.00269 (C_7_H_3_O_4_) | 0.1485 ±0.0016^(d)^ |
| **13** | 14.41 | Isobutyryl-tricaffeoylhexaric acid isomer | C_37_H_34_O_18_ | 765.16669 | 765.16732 | -0.82 | [M − H − 2caffeoyl]^−^  [M − H − 3caffeoyl]^−^  [M − H − 3caffeoyl − H_2_O]^−^  [M − H − 2caffeoyl − hexA − iBT]^−^  [M − H − 3caffeoyl − C_4_H_6_O − H_2_O]^−^  [M − H − 2caffeoyl − C_4_H_6_O − H_2_O]^−^ | 441.10422 (C_19_H_21_O_12_) 279.0722 (C_10_H_15_O_9_)  261.06168 (C_10_H_13_O_8_) 179.03389 (C_9_H_7_O_4_) 191.01891 (C_6_H_7_O_7_) 353.05115 (C_15_H_13_O_10_) | 0.01806 ±0.00057^(c)^ |
| **14** | 16.14 | Coumaroyl derivative | C_48_H_52_NO_9_ | 785.35638 | 785.35555 | 1.05 | [M − H − C_8_H_8_O]^−^  [M − H − 2C_8_H_8_O]^−^  [M − H − coumaroyl]^−^  [M − H − 2coumaroyl− C_21_H_34_NO_3_]^−^ | 665.29852 (C_40_H_43_NO_8_)  545.24103 (C_32_H_35_NO_7_)  639.31927 (C_39_H_45_NO_7_)  145.02821 (C_9_H_5_O_2_) | 0.1145 ±0.0026^(f)^ |

Qualitative analysis was performed using LC-Q-Orbitrap HRMS in negative ion mode. The abbreviations used are as follows: caffeoyl, −C_9_H_6_O_3_, *m/z* 162.031; coumaroyl, −C_9_H_6_O_2_, *m/z* 146.037; Hex (hexose), −C_6_H_10_O_5_, *m/z* 162.053; hexA (hexaric acid), −C_6_H_8_O_7_, *m/z* 192.027; iBt (isobutyryl), −C_4_H_6_O, *m/z* 70.042; malonyl, −C_3_H_2_O_3_, *m/z* 86.000; quinoyl, −C_7_H_10_O_5_, *m/z* 174.052. Quantitative analysis was performed based on LC-PAD analyses and the content of individual analytes was calculated based: (a) on authentic standards; (b) as chlorogenic acid equivalents; (c) as caffeic acid equivalents; (d) as quercetin equivalents; (e) as kaempferol equivalents; (f) as *p*-coumaric acid equivalents. The data are expressed as mean values of three independent experiments ± standard deviation. Basic validation parameters (external calibration; linear regression): chlorogenic acid: linear range, 12–122 µg mL⁻¹; standard curve, *y* = 77373*x* + 34987; *r^2^*= 0.992; LOD, 0.07 µg mL⁻¹; LOQ, 0.25 µg mL⁻¹, caffeic acid: linear range, 12–122 µg mL⁻¹; standard curve, *y* = 96716*x* + 43733; *r^2^*= 0.992; LOD, 0.06 µg mL⁻¹; LOQ 0.20 µg mL⁻¹, *p*-coumaric acid: linear range, 12–122 µg mL⁻¹; standard curve, *y* = 125731*x* + 56853; *r^2^*= 0.997; LOD, 0.06 µg mL⁻¹; LOQ, 0.20 µg mL⁻¹, quercetin: linear range, 7–70 µg mL⁻¹; standard curve, *y* = 47783*x* - 28307; *r^2^*= 0.991; LOD, 0.12 µg mL⁻¹; LOQ, 0.40 µg mL⁻¹, kaempferol: linear range, 12–117 µg mL⁻¹; standard curve, *y* = 65421*x* - 67453; *r^2^*= 0.997; LOD, 0.09 µg mL⁻¹; LOQ, 0.29 µg mL⁻¹. *Notes:* *x* — analyte concentration (µg mL⁻¹); *y* — peak area at the detection wavelength.

**Sup.table 3.** Retention time (RT, min), proposed formula, theoretical *m/z* value of the precursor ion (Da), experimental *m/z* value of the precursor ion (Da), accuracy (Δm, ppm), *m/z* values and formulae of diagnostic fragment ions (Da) and content (mg/g DW) of major compounds identified in *Rumex obtusifolius* extracts.

| **No** | **RT [min]** | **Tentative Identification** | **Formula** | **Theoretical**  **[M − H]^−^** | **Experimental**  **[M − H]^−^** | **Δm [ppm]** | **Product ions** | | **Content (mg/g DW)** |
| --- | --- | --- | --- | --- | --- | --- | --- | --- | --- |
|  |  |  |  |  |  |  | **Neutral-loss** | ***m/z* (formula)** |  |
| **1** | 3.33 | Procyanidin trimer | C_45_H_38_O_18_ | 865.19799 | 865.19873 | -0.85 | [M − H − 2(epi)Cat − 2H]^−^  [M − H − (epi)Cat − 2H]^−^  [M − H − C_8_H_8_O_3_ − H_2_O]^−^  [M − H − (epi)Cat]^−^  [M − H − (epi)Cat − C_9_H_8_O_3_ ]^−^  [M − H − 2(epi)Cat − C_9_H_8_O_3_]^−^  [M − H − (epi)Cat − C_8_H_8_O_3_ ]^−^  [M − H − 2(epi)Cat]^−^  [M − H − C_8_H_8_O_3_]^−^ | 287.05624 (C_15_H_11_O_6_)  575.12006 (C_30_H_23_O_12_)  695.14124 (C_37_H_27_O_14_)  577.13562 (C_30_H_25_O_12_)  413.08804 (C_21_H_17_O_9_)  125.02319 (C_6_H_5_O_3_)  425.0885 (C_22_H_17_O_9_)  289.07193 (C_15_H_13_O_6_)  713.15381 (C_37_H_29_O_15_) | 0.259  ±0.031^(b)^ |
| **2** | 5.75 | Protocatechuic acid | C_7_H_6_O_4_ | 153.01878 | 153.01900 | -1.44 | [M − H − CHO_2_ ]^−^  [M − H − CO_2_]^−^  [M − H − CO_2_ − H_2_O]^−^ | 108.02043 (C_6_H_4_O_2_)  109.02849 (C_6_H_5_O_2_)  91.01759 (C_6_H_3_O) | 0.049  ±0.012^(a)^ |
| **3** | 6.07 | Procyanidin dimer | C_30_H_26_O_12_ | 577.13460 | 577.13523 | -1.09 | [M − H − (epi)Cat]^−^  [M − H − C_8_H_8_O_3_ − H_2_O]^−^  [M − H − (epi)Cat − C_9_H_8_O_3_]^−^  [M − H − (epi)Cat − C_6_H_8_O_3_]^−^  [M − H − (epi)Cat − CO_2_ ]^−^  [M − H − C_8_H_8_O_3_]^−^  [M − H − C_11_H_10_O_6_]^−^ | 289.07193 (C_15_H_13_O_6_)  407.07727 (C_22_H_15_O_8_)  125.0232 (C_6_H_5_O_3_)  161.02347 (C_9_H_5_O_3_)  245.08188 (C_14_H_13_O_4_)  425.08774 (C_22_H_17_O_9_)  339.08734 (C_19_H_15_O_6_) | 0.148  ±0.038^(b)^ |
| **4** | 6.72 | Procyanidin tetramer one A-type linkage | C_60_H_48_O_24_ | 1151.24573 | 1151.24685 | -0.97 | [M − H − (epi)Cat]^−^  [M − H −(epi)Cat − C_8_H_8_O_3_]^−^  [M − H − C_8_H_8_O_3_]^−^  [M − H − 2(epi)Cat − 2H]^−^ | 863.18359 (C_45_H_35_O_18_)  711.13635 (C_37_H_27_O_15_)  999.19971 (C_52_H_39_O_21_)  573.10443 (C_30_H_21_O_12_) | 0.304  ±0.049^(b)^ |
| **5** | 7.05 | Procyanidin dimer | C_30_H_26_O_12_ | 577.13460 | 577.13522 | -1.07 | [M − H − (epi)Cat]^−^  [M − H − C_8_H_8_O_3_ − H_2_O]^−^  [M − H − (epi)Cat − C_9_H_8_O_3_]^−^  [M − H − (epi)Cat − C_6_H_8_O_3_]^−^  [M − H − (epi)Cat − CO_2_ ]^−^  [M − H − C_8_H_8_O_3_]^−^  [M − H − C_11_H_10_O_6_ ] | 289.07202 (C_15_H_13_O_6_)  407.07733 (C_22_H_15_O_8_)  125.02323 (C_6_H_5_O_3_)  161.02347 (C_9_H_5_O_3_)  245.08188 (C_14_H_13_O_4_)  425.08887 (C_22_H_17_O_9_)  339.08765 (C_19_H_15_O_6_) | 0.208  ±0.028^(b)^ |
| **6** | 7.64 | (+)-Catechin | C_15_H_14_O_6_ | 289.07121 | 289.07151 | -1.04 | [M − H − C_9_H_8_O_4_]^−^  [M − H − C_8_H_6_O_4_]^−^  [M − H − C_9_H_8_O_3_]^−^  [M − H − C_7_H_6_O_3_]^−^  [M − H − C_8_H_8_O_3_]^−^ | 109.02829 (C_6_H_5_O_2_)  123.04402 (C_7_H_7_O_2_)  125.02322 (C_6_H_5_O_3_)  151.03897 (C_8_H_7_O_3_)  137.02347 (C_7_H_5_O_3_) | 0.462  ±0.039^(a)^ |
| **7** | 8.51 | Procyanidin dimer | C_30_H_26_O_12_ | 577.13460 | 577.13525 | -1.13 | [M − H − (epi)Cat]^−^  [M − H − C_8_H_8_O_3_ − H_2_O]^−^  [M − H − (epi)Cat − C_9_H_8_O_3_]^−^  [M − H − (epi)Cat − C_6_H_8_O_3_]^−^  [M − H − (epi)Cat − CO_2_ ]^−^  [M − H − C_8_H_8_O_3_]^−^ | 289.07202 (C_15_H_13_O_6_)  407.07727 (C_22_H_15_O_8_)  125.02322 (C_6_H_5_O_3_)  161.0235 (C_9_H_5_O_3_)  245.08191 (C_14_H_13_O_4_)  425.08875 (C_22_H_17_O_9_) | 1.196  ±0.030^(b)^ |
| **8** | 9.40 | Epicatechin | C_15_H_14_O_6_ | 289.07121 | 289.07153 | -1.11 | [M − H − C_9_H_8_O_4_]^−^  [M − H − C_8_H_6_O_4_]^−^  [M − H − C_9_H_8_O_3_]^−^  [M − H − C_7_H_6_O_3_]^−^  [M − H − C_8_H_8_O_3_]^−^  [M − H − C_2_H_2_O − CO_2_]^−^ | 109.02827 (C_6_H_5_O_2_)  123.04401 (C_7_H_7_O_2_)  125.02326 (C_6_H_5_O_3_)  151.03911 (C_8_H_7_O_3_)  137.02341 (C_7_H_5_O_3_)  203.07086 (C_12_H_11_O_3_) | 0.361  ±0.034^(a)^ |
| **9** | 9.87 | Procyanidin tetramer monogallate | C_67_H_54_O_28_ | 1305.27234 | 1305.274935  (652.636  [M − 2H]^2−^) | -1.98 | [M − H − 3(epi)Cat − galloyl − C_9_H_8_O_3_]^−^  [M − H − 3(epi)Cat − galloyl]^−^  [M − H − 3(epi)Cat − C_15_H_12_O_5_]^−^  [M − H − 3(epi)Cat − galloyl − 2H]^−^  [M − H − 3(epi)Cat − galloyl − C_7_H_6_O_3_]^−^  [M − H − 2(epi)Cat − galloyl − C_8_H_8_O_3_ − H_2_O]^−^ | 125.02323 (C_6_H_5_O_3_)  289.07205 (C_15_H_13_O_6_)  169.01343 (C_7_H_5_O_5_)  287.05637 (C_15_H_11_O_6_)  151.03912 (C_8_H_7_O_3_)  407.07706 (C_22_H_15_O_8_) | 0.207  ±0.029^(b)^ |
| **10** | 10.08 | Procyanidin trimer one A-type linkage | C_45_H_36_O_18_ | 863.18234 | 863.18341 | -1.24 | [M − H − (epi)Cat − C_9_H_8_O_3_ ]^−^  [M − H − (epi)Cat − C_15_H_10_O_6_]^−^  [M − H − C_8_H_8_O_3_]^−^  [M − H − (epi)Cat − 2H]^−^ | 411.07242 (C_21_H_15_O_9_)  289.07208 (C_15_H_13_O_6_)  711.13605 (C_37_H_27_O_15_)  573.10400 (C_30_H_21_O_12_) | 1.225  ±0.070^(b)^ |
| **11** | 10.70 | Procyanidin tetramer | C_60_H_50_O_24_ | 1153.26138 | 1153.26208 | -0.61 | [M − H − 2(epi)Cat − 2H]^−^  [M − H − 2(epi)Cat]^−^  [M − H − (epi)Cat − 2H]^−^  [M − H − (epi)Cat]^−^  [M − H − 3(epi)Cat − 2H]^−^  [M − H − C_8_H_8_O_3_]^−^ | 575.12000 (C_30_H_23_O_12_)  577.13550 (C_30_H_25_O_12_)  863.18384 (C_45_H_35_O_18_)  865.19928 (C_45_H_37_O_18_)  287.05630 (C_15_H_11_O_6_)  1001.21448 (C_52_H_41_O_21_) | 0.271  ±0.022^(b)^ |
| **12** | 12.36 | Procyanidin trimer digallate | C_59_H_46_O_26_ | 1169.21991 | 1169.22181 | -1.62 | [M − H − (epi)Cat]^−^  [M − H − C_6_H_6_O_3_]^−^  [M − H − C_3_H_8_O_6_]^−^ | 881.15833 (C_44_H_33_O_20_)  1043.18872 (C_53_H_39_O_23_)  1017.18872 (C_55_H_37_O_20_) | 0.987  ±0.031^(b)^ |
| **13** | 12.92 | Procyanidin dimer digallate | C_44_H_34_O_20_ | 881.15652 | 881.15756 | -1.18 | [M − H − galloyl]^−^  [M − H − 2galloyl − C_8_H_8_O_3_ − H_2_O]^−^  [M − H − 2galloyl − H_2_O]^−^  [M − H − (epi)Cat − 2galloyl − 2H]^−^  [M − H − galloyl − H_2_O]^−^  [M − H − 2galloyl − 2H_2_O]^−^  [M − H − − (epi)Cat − 2galloyl − H_2_O − 2H]^−^ | 729.14703 (C_37_H_29_O_16_)  407.07748 (C_22_H_15_O_8_)  559.12952 (C_30_H_23_O_11_)  287.05630 (C_15_H_11_O_6_)  711.13635 (C_37_H_27_O_15_)  541.11499 (C_30_H_21_O_10_)  269.04575 (C_15_H_9_O_5_) | 0.918  ±0.019^(b)^ |
| **14** | 13.39 | Quercetin-*O*-hexoside | C_21_H_20_O_12_ | 463.08765 | 463.08811 | -0.99 | [M − H − Hex(C_6_H_10_O_5_)]^−^ | 301.03412 (C_15_H_9_O_7_) | 0.1338  ±0.0081^(c)^ |
| **15** | 13.62 | (epi)Catechin monogallate | C_22_H_18_O_10_ | 441.08217 | 441.08260 | -0.97 | [M − H − C_15_H_12_O_5_]^−^  [M − H − galloyl − C_9_H_8_O_3_]^−^  [M − H − galloyl]^−^  [M − H − galloyl − CO_2_]^−^  [M − H − galloyl − C_8_H_8_O_3_]^−^ | 169.01344 (C_7_H_5_O_5_)  125.02326 (C_6_H_5_O_3_)  289.07202 (C_15_H_13_O_6_)  245.08185 (C_14_H_13_O_4_)  137.02338 (C_7_H_5_O_3_) | 0.676  ±0.039^(b)^ |
| **16** | 13.70 | Procyanidin trimer trigallate | C_66_H_50_O_30_ | 1321.23087 | 1321.23280  (660.616 [M − 2H)^2−^] | -1.46 | [M − H − 2galloyl − 2(epi)Cat − C_15_H_12_O_5_]^−^  [M − H − 3galloyl − 2(epi)Cat − C_9_H_8_O_3_]^−^  [M − H − 3galloyl − 2(epi)Cat]^−^  [M − H − 3galloyl − 2(epi)Cat − 2H]^−^  [M − H − 2(epi)Cat − 3galloyl − C_7_H_6_O_3_]^−^  [M − H − 3galloyl − (epi)Cat − C_8_H_8_O_3_ − H_2_O]^−^ | 169.01347 (C_7_H_5_O_5_)  125.02328 (C_6_H_5_O_3_)  289.07205 (C_15_H_13_O_6_)  287.05649 (C_15_H_11_O_6_)  151.03908 (C_8_H_7_O_3_)  407.0777 (C_22_H_15_O_8_) | 0.651  ±0.033^(b)^ |
| **17** | 14.17 | Procyanidin tetramer one A-type linkage | C_60_H_48_O_24_ | 1151.24573 | 1151.24708 | -1.17 | [M − H − 2(epi)Cat]^−^  [M − H − 2(epi)Cat − C_8_H_8_O_3_]^−^  [M − H − 2(epi)Cat − C_6_H_6_O_3_]^−^  [M − H − 2(epi)Cat − 2H]^−^  [M − H − 2(epi)Cat − C_15_H_10_O_6_]^−^  [M − H − (epi)Cat]^−^ | 575.12006 (C_30_H_23_O_12_)  423.07266 (C_22_H_15_O_9_)  449.08835 (C_24_H_17_O_9_)  577.12854 (C_30_H_25_O_12_)  289.07208 (C_15_H_13_O_6_)  863.18414 (C_45_H_35_O_18_) | 0.344  ±0.025^(b)^ |
| **18** | 14.73 | Quercetin-acetyl-hexoside | C_23_H_22_O_13_ | 505.09822 | 505.09880 | -1.15 | [M − H − acyl − Hex]^−^  [M − H − acyl]^−^ | 301.03461 (C_15_H_9_O_7_)  463.08908 (C_21_H_19_O_12_) | 0.0629  ±0.0050^(c)^ |
| **19** | 15.25 | Quercetin-*O*-pentoside | C_20_H_18_O_11_ | 433.07709 | 433.07754 | -1.05 | [M − H − Pent]^−^ | 301.03470 (C_15_H_9_O_7_) | 0.0318  ±0.0028^(c)^ |
| **20** | 15.70 | Procyanidin dimer monogallate | C_37_H_30_O_16_ | 729.14556 | 729.14654 | -1.34 | [M − H − galloyl − C_8_H_8_O_3_ − H_2_O]^−^  [M − H − galloyl − (epi)Cat]^−^  [M − H − galloyl − C_6_H_6_O_3_]^−^  [M − H − galloyl − (epi)Cat − C_9_H_8_O_3_]^−^  [M − H − (epi)Cat − C_15_H_12_O_5_]^−^ | 407.07736 (C_22_H_15_O_8_)  289.07196 (C_15_H_13_O_6_)  451.10419 (C_24_H_19_O_9_)  125.02318 (C_6_H_5_O_3_)  169.01335 (C_7_H_5_O_5_) | 0.773  ±0.043^(b)^ |
| **21** | 17.30 | Procyanidin dimer digallate | C_44_H_34_O_20_ | 881.15652 | 881.15754 | -1.16 | [M − H − galloyl]^−^  [M − H − 2galloyl − C_8_H_8_O_3_ − H_2_O]^−^  [M − H − 2galloyl − H_2_O]^−^  [M − H − galloyl − H_2_O]^−^  [M − H − 2galloyl − 2H_2_O]^−^  [M − H − (epi)Cat − 2galloyl − 2H]^−^ | 729.14709 (C_37_H_29_O_16_)  407.07742 (C_22_H_15_O_8_)  559.13049 (C_30_H_23_O_11_)  711.13593 (C_37_H_27_O_15_)  541.11487 (C_30_H_21_O_10_)  287.05621 (C_15_H_11_O_6_) | 1.426  ±0.030^(b)^ |

Qualitative analysis was performed using LC-Q-Orbitrap HRMS in negative ion mode. The abbreviations used are as follows: Acyl, −C_2_H_2_O, *m/z* 42.010; (epi)Cat, (epicatechin/catechin), −C_15_H_12_O_6_, *m/z* 288.063; galloyl, −C_7_H_4_O_4_, *m/z* 152.011; Hex (hexose), −C_6_H_10_O_5_, *m/z* 162.053; Pent (pentose), −C_5_H_8_O_4_, *m/z* 132.042. Quantitative analysis was performed based on LC-PAD analyses and the content of individual analytes was calculated based: (a) on authentic standards; (b) as catechin equivalents; (c) as quercetin equivalents. The data are expressed as mean values of three independent experiments ± standard deviation. Basic validation parameters (external calibration; linear regression): protocatechuic acid: linear range, 50–400 µg mL⁻¹; standard curve, *y* = 3.454*x* - 1.536; *r^2^*= 0.999; LOD, 4.34 µg mL⁻¹; LOQ, 14.47 µg mL⁻¹, (+)-catechin: linear range, 50–400 µg mL⁻¹; standard curve, *y* = 0.881*x* + 0.357; *r^2^*= 0.999; LOD, 17.02 µg mL⁻¹; LOQ 56.74 µg mL⁻¹, (-)-epicatechin: linear range, 47–380 µg mL⁻¹; standard curve, *y* = 0.731*x* + 0.964; *r^2^*= 0.999; LOD, 20.53 µg mL⁻¹; LOQ, 68.44 µg mL⁻¹, quercetin: linear range, 50–400 µg mL⁻¹; standard curve, *y* = 3.917*x* - 30.500; *r^2^*= 0.999; LOD, 3.83 µg mL⁻¹; LOQ, 12.77 µg mL⁻¹. *Notes:* *x* — analyte concentration (µg mL⁻¹); *y* — peak area at the detection wavelength.
